# The value of manual backward contact tracing to control COVID-19 in practice, the Netherlands, February to March 2021: a pilot study

**DOI:** 10.2807/1560-7917.ES.2023.28.41.2200916

**Published:** 2023-10-12

**Authors:** Timo Louis Boelsums, Inge Anna Theresia van de Luitgaarden, Jane Whelan, Hanna Poell, Charlotte Maria Hoffman, Ewout Fanoy, Maaike Buskermolen, Jan Hendrik Richardus

**Affiliations:** 1 Department of Infectious Disease Control, Public Health Service Rotterdam-Rijnmond, Rotterdam, the Netherlands; 2 Department of Infectious Disease Control, Public Health Service Amsterdam-Amstelland, Amsterdam, the Netherlands; 3 Department of Public Health, Erasmus MC, University Medical Center Rotterdam, Rotterdam, the Netherlands

**Keywords:** COVID-19, SARS-CoV-2, backward contact tracing, pandemic preparedness, public health response, infectious disease control

## Abstract

**Background:**

Contact tracing has been a key component of COVID-19 outbreak control. Backward contact tracing (BCT) aims to trace the source that infected the index case and, thereafter, the cases infected by the source. Modelling studies have suggested BCT will substantially reduce SARS-CoV-2 transmission in addition to forward contact tracing.

**Aim:**

To assess the feasibility and impact of adding BCT in practice.

**Methods:**

We identified COVID-19 cases who were already registered in the electronic database between 19 February and 10 March 2021 for routine contact tracing at the Public Health Service (PHS) of Rotterdam-Rijnmond, the Netherlands (pop. 1.3 million). We investigated if, through a structured questionnaire by dedicated contact tracers, we could trace additional sources and cases infected by these sources. Potential sources identified by the index were approached to trace the source’s contacts. We evaluated the number of source contacts that could be additionally quarantined.

**Results:**

Of 7,448 COVID-19 cases interviewed in the study period, 47% (n = 3,497) indicated a source that was already registered as a case in the PHS electronic database. A potential, not yet registered source was traced in 13% (n = 979). Backward contact tracing was possible in 62 of 979 cases, from whom an additional 133 potential sources were traced, and four were eligible for tracing of source contacts. Two additional contacts traced had to stay in quarantine for 1 day. No new COVID-19 cases were confirmed.

**Conclusions:**

The addition of manual BCT to control the COVID-19 pandemic did not provide added value in our study setting.

**Key public health message**

**What did you want to address in this study?**

In the early stages of the COVID-19 pandemic, public health authorities used forward contact tracing as a tool to curb the spread, actively searching for individuals exposed to an infected person. Mathematical models indicate that 'backward contact tracing' (BCT), an approach targeting the potential sources of an infection to trace additional contacts, can be a valuable complement. We aimed to examine the practical application and real-world impact of BCT.

**What have we learnt from this study?**

We found that implementing manual BCT in addition to our standard contact tracing methods did not substantially disrupt SARS-CoV-2 transmission chains. Notably, the implementation of BCT, particularly in a manual context, proved to be resource-intensive. The promising outcomes of mathematical models have not translated readily into tangible benefits in a real-world setting.

**What are the implications of your findings for public health?**

Our findings underscore the importance of investigating a range of methods to improve contact tracing, especially with new virus variants. The challenges of implementing BCT highlight the need to balance theoretical promise with practical constraints in tracing strategies.

## Introduction

From the onset of the pandemic up to 1 September 2023, over 770 million cases and 6.9 million deaths have been attributed to coronavirus disease (COVID-19) worldwide [1]. Apart from the health risks of the infection on the individual, the pandemic and the measures implemented to control it, e.g. lockdowns, substantially affected society as a whole [2].

Testing and contact tracing are key strategies for containment of COVID-19. Forward contact tracing is the process of identifying people who have been in contact with a confirmed case (hereafter, the index case) during the case’s infectious period in order to isolate or quarantine them. Forward tracing is commonly conducted for epidemiological investigations and has been the primary focus of many protocols for contact tracing of COVID-19. Further viral transmission is then reduced by quarantining asymptomatic (potentially presymptomatic) individuals, as well as testing and isolating symptomatic contacts [3-6].

**Figure 1:**
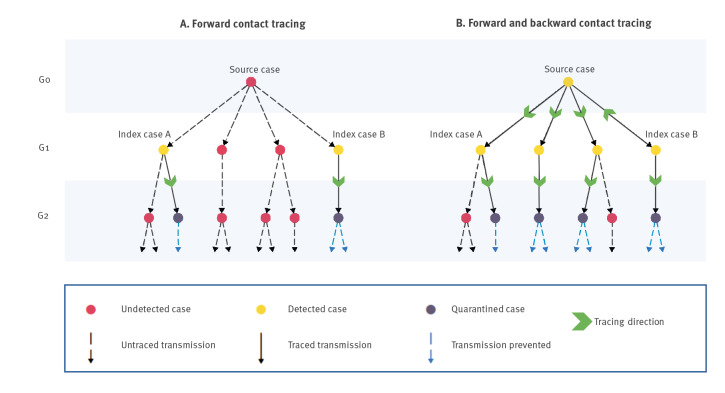
Graphical representation of forward and backward contact tracing

G0: generation 0; G1: generation 1; G2: generation 2.

Backward contact tracing (BCT) is an addition to forward contact tracing, which aims to trace the source that infected the index case, followed by forward contact tracing from that source case (Figure 1). This could increase the impact of contact tracing, since more contacts at risk of spreading the virus may be traced and subsequently quarantined. Through this process, additional transmission chains originating from the source case can be identified: a source case (generation 0; G0) may have infected, next to the index case, additional contacts (generation 1; G1), which in turn may have infected even more other contacts (generation 2; G2).

The vertical axis represents the generations of viral transmission, from the source case (G0) to the index case (G1) and cases infected by the index case (G2). When compared to forward contact tracing only (A), application of backward contact tracing in addition to forward contact tracing (B) may interrupt more transmission chains (blue arrows) and may prevent more infections. This figure was adapted from Endo et al. [14].

**Figure 2:**
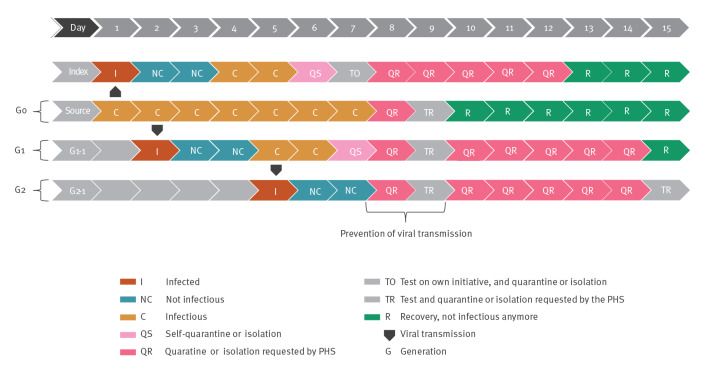
Example of viral transmission between the source, generation-1 and generation-2 contacts

For BCT in addition to forward contact tracing to have more impact than forward contact tracing alone, both characteristics of the disease and the contact tracing process need to meet certain requirements. Regarding the disease characteristics, one crucial requirement according to modelling studies is that the spread displays individual-level variation in the number of secondary transmissions per index, i.e. overdispersion: while the majority of individuals infect none or few other persons, a small minority infects many (during superspreading events) [7]. A consequence is that the source case who has infected the index case is likely to have infected a higher number of people than the index case has. This overdispersion has been observed in several COVID-19 studies [8-13]. Regarding contact tracing, a crucial requirement is that it should be feasible to contact the source and additional contacts in a timely manner. The additional contacts traced should have only recently contracted the virus or be close to the beginning of their infectious period, and not yet be infectious (i.e. at least 48 h before onset of symptoms), so that if they are traced and quarantined in time, the viral transmission chain can be interrupted (Figure 2).

PHS: Public Health Service.

By identifying the potential source of the index case and quarantining the G1 and G2 contacts of this source as soon as they are identified, the viral transmission chain(s) may be interrupted. In contrast, if G2 contacts are not identified and quarantined in time, viral transmission may continue.

Several modelling studies suggest that digital or manual BCT in addition to forward contact tracing has more impact to control the pandemic, compared with forward contact tracing only [14-17]. One empirical study, by Raymenants et al. performed during the COVID-19 pandemic, examines BCT among students in the Belgian city of Leuven [18]. In this study, the contact tracing window was extended from 2 days (start of infectious period of index case) to 7 days before symptom onset to trace source (G0) cases and additional G1 contacts. The investigators identified an additional 42% COVID-19 cases. However, whether identification of potential sources effectively leads to interruption of viral transmission chains by quarantining additional contacts at risk has not yet been studied in practice. 

To assess the feasibility and impact of BCT in a real-world setting, we performed a pilot study in the Rotterdam-Rijnmond area of the Netherlands during February and March 2021. During this time, there was a partial lockdown and the severe acute respiratory syndrome coronavirus 2 (SARS-CoV-2) Alpha variant of concern (Phylogenetic Assignment of Named Global Outbreak (Pango) lineage designation B.1.1.7) was the dominant circulating variant. The study was conducted in two steps, where the first step involved identifying potential sources through regular forward contact tracing, and the second step involved implementing BCT to identify additional contacts at risk.

## Methods

### Study setting

During the pilot study period of February and March 2021, a partial lockdown was in effect in the Netherlands. Most relevant characteristics of the pandemic circumstances of that period, including lockdown measures, are summarised in the Table.

**Table:** Characteristics of the COVID-19 pandemic during the pilot study, the Netherlands, 19 February–10 March 2021

Variables	Value	Source
Dominant SARS-CoV-2 strain	Alpha variant (Pango lineage designation B.1.1.7)	[22]
Estimated basic reproduction number (R_0_)^a^	0.97–1.17	[20]
Total number of COVID-19 cases per day	3,808–5,256	[20]
Hospital admissions for COVID-19 per day, mean (range)	174 (123–240)^b^	[20]
Test recommendations	Individuals with symptoms indicative of COVID-19 were strongly advised to test for SARS-CoV-2 at a local Public Health Service testing site. Self-testing was not yet available.	[19]
Vaccination coverage^c^		[23]
Partly vaccinated	7%	
Fully vaccinated	3%	
Lockdown measures		[24]
Education	All education facilities were closed, excluding primary schools. From 1 March 2021, secondary schools were allowed to open under restrictions.	
Shops	Only shops selling essential products (such as supermarkets and pharmacies) were open; all others were closed. From 1 March 2021, shops were allowed to open for the pick-up of online orders; hairdressers and similar professions could have clients.	
Household visits	Inviting > 1 person at home outside the household was discouraged.	
Restaurants and cafes	All were closed.	
Sports	Organised sports activities were not allowed. From March 2021, individuals ≤ 27 years old were allowed to practice sports together.	

Pango: Phylogenetic Assignment of Named Global Outbreak; SARS-CoV-2: severe acute respiratory syndrome coronavirus 2.

^a^ Expected number of cases directly generated by one case.

^b^ The range during the period of October 2020–April 2023 was 4–364.

^c^ The vaccination campaign in the Netherlands started on 6 January 2021. Those vaccinated at the time of the pilot study were mainly older individuals in nursing homes (aged ≥ 65 years) and healthcare workers (aged 18–66 years). Partly vaccinated: individuals that had received one vaccination with Comirnaty (BNT162b2 mRNA, BioNTech-Pfizer), Spikevax (mRNA-1273, Moderna) or Vaxzevria (ChAdOx1 nCoV-19, Oxford-AstraZeneca); fully vaccinated: individuals that had received two doses.

There were no region-specific epidemiological data available, but we assumed that the pandemic situation in Rotterdam-Rijnmond area, with a population size of 1.3 million inhabitants, was largely similar to the situation in the whole country (pop. 17 million). The personnel capacity slightly exceeded the demand for the routine contact tracing at the Public Health Service (PHS) Rotterdam-Rijnmond, enabling the implementation of this pilot study without delaying the routine contact tracing.

### Routine contact tracing programme

In the Netherlands, contact tracing for SARS-CoV-2 infections is a task of the PHS. All individuals who tested positive for SARS-CoV-2 were mandatorily notified to the PHS by the laboratories after a positive reverse transcription PCR (RT-PCR) result at a local PHS testing site. A contact tracer then performed the contact tracing by structured interviews over the phone. This interview aimed to inform the index case about the infection and to identify contacts at risk. The index case was strongly advised to isolate for 7 days after symptom onset or longer, until they were symptom-free for 24 h (isolation period). If the index case underwent testing despite being asymptomatic, 3 days of isolation was advised upon a positive test result. Should any symptoms subsequently appear, the index case was advised to prolong the isolation to 7 days.

Forward traced contacts at risk were advised to quarantine to prevent further spread of the infection for 10 days (quarantine period), in accordance with the national guidelines [19]. Index cases were asked a single question to identify the potential source case, defined as an individual who was considered likely to be the source of the infection of the index case, as judged by the index case. All contact tracing data were stored in an electronic patient file system, hereafter referred to as the electronic database. All 25 PHS in the Netherlands have, although limited, access to the electronic database of every other PHS; name and date of birth can be checked. 

### Backward contact tracing

In our study, we define BCT as the combination of source tracing backwards from the index case to identify additional potential source cases and the consecutive forward tracing from the source case. All contact tracers were instructed to ask the index case explicitly for potential sources through more detailed questioning than during the routine contact tracing. The pilot study evaluation was composed of quantitative and qualitative methods and performed in two steps, which are outlined in Supplement S1.

#### Source identification through routine contact tracing team

During routine contact tracing, index cases received one question concerning the potential source of their infection. Contact tracers would evaluate the 7-day period before disease onset with the index case day by day if the index case could not immediately think of a potential source. If the index case thought they could indicate the source of their infection, the contact details of the potential source were registered in the electronic database. Only if enough contact details were provided, e.g. name and one of the following: date of birth or connection to a registered COVID-19 outbreak, was it possible to check the database to determine whether this person had already been known to the PHS as a recent COVID-19 case. If the source was already registered, the source case was linked in the database to the index case. Potential sources who could not be identified in the database were not contacted during routine contact tracing.

#### Backward contact tracing through dedicated team

For the implementation of the second part of the pilot study, we formed a dedicated contact tracing team of 15 contact tracers, who received a dedicated training of 2 h (hereafter, the dedicated contact tracers). To perform BCT, they followed step-by-step instructions, which are outlined in Dutch in Supplement S2. We expanded the standard questionnaire with additional questions to trace the potential source of infection. All index cases for which the potential source could not be identified in the electronic database were further investigated by the dedicated contact tracing team.

The dedicated contact tracers followed a structured approach to identify potential sources during the routine interview with the index case: they classified contacts of the index case as potential sources if they had been in close contact with the index case (> 15 min within 1.5 m proximity) during the period the transmission of the virus could have taken place (7 to 2 days before disease onset of the index case). If the potential source was already registered as a COVID-19 case in the PHS electronic database and contact tracing was performed for this person, the source was excluded from further study as no additional action from the PHS was required.

Consent from the index case to contact the potential source(s) was obtained by the dedicated contact tracers. Subsequently, they contacted the potential source to (i) verify time of contact with index case and (ii) identify the infectious period (2 days before disease onset until 7 days after disease onset and 24 h free of symptoms). If potential sources had been asymptomatic, they were excluded from further study since no infectious period could be identified. In case an infectious period could be identified, the dedicated contact tracers prompted the potential source to undergo a SARS-CoV-2 test at a PHS testing site and identified their contacts during the infectious period. These contacts of the potential source were indicated as G1 contacts (Figure 1).

The dedicated contact tracers approached the G1 contacts. They advised symptomatic G1 contacts to test, and G2 contacts were traced. The dedicated contact tracers approached G2 contacts and advised them to quarantine irrespective of symptoms (Figure 2). Afterwards, the experience of the dedicated contact tracers in terms of time invested was determined through unstructured face-to-face interviews with the study team.

### Data analysis

For the first step of this pilot study, we evaluated the number of sources who were already registered in the electronic database and the setting where the viral transmission had taken place. For the second step of our study, we assessed the feasibility and impact of manual BCT.

To assess feasibility, we determined the number of index cases with one or more potential sources and, if available, the setting where the source infected the index case, the median number of potential sources per index case, and the number and proportion of G1 and G2 contacts that were traced and contacted. We estimated the time needed to perform BCT per case. We collected reasons for not succeeding to trace a source from the dedicated contact tracing team.

To assess the impact, we determined the number and proportion of potential sources and G1 contacts with a positive test result, the number of traced G2 contacts and the mean number of days that quarantine was advised. To assess the burden, we evaluated the duration that it took the dedicated contact tracers to perform the BCT and the number of quarantine days for contacts of potential sources who were negative for SARS-CoV-2 after testing.

## Results

### Source identification during routine contact tracing process

From 19 February to 10 March 2021, the PHS of Rotterdam-Rijnmond received a total of 7,448 notifications of cases who tested positive for SARS-CoV-2.

**Figure 3:**
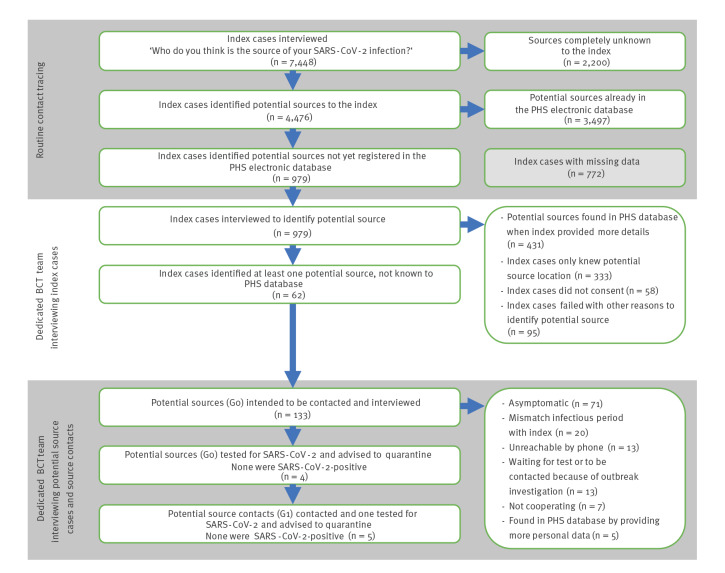
Flow diagram of backward contact tracing with outcomes, the Netherlands, 19 February–10 March 2021

BCT: backward contact tracing; PHS: Public Health Service; SARS-CoV-2: severe acute respiratory syndrome coronavirus 2.

Of the 7,448 cases interviewed, 47% (n = 3,497) indicated an index case-reported potential source case of the SARS-CoV-2 infection who was already recently registered as SARS-CoV-2-positive in the PHS electronic database (Figure 3). Of those 3,497 sources, the suspected setting in which these viral transmissions occurred were: at home (48%, n = 1,686), during a visit (18%, n = 630), at work (15%, n = 527), at school (8%, n = 280), in a nursing home (3%, n = 122) or other (7%, n = 252). In 13% of 7,448 cases (n = 979), the contact tracers identified potential sources who were not yet registered in the PHS electronic database. In all other instances, the index case could not indicate a potential source (30%, n = 2,200) or the contact tracer did not record whether the index case could indicate a source (missing data, 10%, n = 772). The median duration from disease onset in symptomatic index cases to testing and from testing to first contact between index case and a contact tracer in these cases were both 2 days, and the median duration from disease onset to contact between index case and contact tracer was 4 days.

### Backward contact tracing by the dedicated contact tracing team

The dedicated contact tracers investigated the 979 files where the index case had reported potential new source cases. They found that most cases were not eligible for BCT, as described in Figure 3. Of the index case-reported potential sources in these files 44% (n = 431) were already registered in the PHS electronic database, but had not yet been linked to the index case. Because of incomplete details of the potential source case, the routine contact tracers were previously not able to identify these sources in the electronic database. In other cases (34%, n = 333), the index case could only indicate a potential source location but no specific person. In a minority, the index did not give permission for further investigation (6%, n = 58) or contacting the potential source case was not possible (10%, n = 95) (Figure 3). Eventually, 62 index cases (6%) with 133 potential source cases were eligible for BCT. The median number of potential sources per index case was 1.5 (range: 1–10). Including the additional identified sources (n = 431) in this step, in total 53% (3,928/7,448) of all sources were known to the PHS.

Of the 133 potential sources contacted, 97% (n = 129) were ineligible for further tracing of G1 contacts. The main reasons were that (i) the potential source had not shown signs of COVID-19 in the last 14 days (53%, n = 71), or that (ii) the dedicated contact tracer concluded that SARS-CoV-2 transmission from the potential source to the index case could not have taken place (15%, n = 20) because the potential source tested SARS-CoV-2-negative or the infectious period of the potential source did not match with the period the index could have contracted the virus. In other instances, the dedicated team could not succeed with BCT because: they were unable to reach the source by phone after multiple attempts (10%, n = 13), the potential source was already registered in the electronic database and retrieved based on further personal details provided by the potential source (4%, n = 5), the potential source did not cooperate (5%, n = 7), the potential source was waiting for the result of a SARS-CoV-2 test or was part of an COVID-19 outbreak investigation (10%, n = 13) (Figure 3).

For the remaining four potential sources, the dedicated contact tracers identified and contacted five G1 contacts, of whom one had symptoms of COVID-19. They contacted the G2 contacts of this person and advised these contacts to quarantine. All four potential sources and the symptomatic G1 contact tested negative for SARS-CoV-2 and the G2 contacts were relieved from quarantine. The median number of days for which quarantine was advised to G2 contacts was 1 day, since testing results from G1 contacts were known after 1 day. The complete process of identifying and contacting the potential source and their G1 and G2 contacts, took a trained contact tracer 1 additional working day on average per index case.

## Discussion

Mathematical modelling has suggested a promising theoretical opportunity to improve contact tracing of COVID-19, through the addition of BCT. In our study, we demonstrate the practical implementation of manual BCT to control COVID-19 when applied in real-world, public health practice. Backward contact tracing ultimately had limited yield, with considerable resource implications.

Contact tracing in practice is a complex process, relying on the cases’ ability to retrieve the potential sources of their SARS-CoV-2 infection and their willingness to share this personal information, but also on the expertise of the contact tracers conducting the interviews. To correctly assess the added value of BCT in practice, we specifically trained contact tracers in additional interviewing techniques, to lower the risk of missing potential sources during source tracing. From a large sample of COVID-19 cases, we made numerous attempts to contact each index, source and contacts to enhance participation.

Despite these efforts, we could not identify additional COVID-19 cases or contacts with this method. We were not able to interrupt any SARS-CoV-2 transmission chain, while imposing extra time and efforts for the contact tracers, and inconvenience for all additionally contacted individuals. Our results were not in line with the projections of the mathematical models [14-17]. We suggest four main explanations for the observed discrepancies. Firstly, we could retrieve fewer additional potential sources by manual BCT than was suggested in modelling studies. In over 50% of the regular contact tracing interviews that were performed during our study, the potential source was already registered as a COVID-19 case and was therefore already known to the PHS. Of note, directions of SARS-CoV-2 transmission are difficult to determine, index case-reported potential source cases could in fact also be G1 contacts, which could have led to an overestimation of source cases identified. In another 30% of the interviews, the potential source remained unidentified. Moreover, in most cases with a previously unknown potential source, the dedicated team did not succeed in further BCT. For comparison, from one modelling study where we could extract a probability of identifying the source (G0) case by BCT, this was 50 to 80% [14]. From other models, we could not extract any corresponding parameter values [15-17].

Secondly, inclusion of asymptomatic potential sources was challenging in practice. In reality, their infectious period is unknown, unlike in a modelled situation where it can be assumed [15]. In our pilot study, more than 50% of the potential sources contacted by the dedicated team were asymptomatic. In this circumstance, we considered BCT of asymptomatic potential sources and quarantining their contacts too burdensome and therefore excluded asymptomatic potential sources. However, we expect that this would not have changed our overall conclusion.

Thirdly, in most modelling studies, a realistic representation of social structures, i.e. the underlying societal patterns and behaviours, seems to be lacking or incomplete [13,15-17]. The mathematical models assume a clear distinction between forward and backward tracing, but in practice, we found these concepts to be intertwined considering other social factors. For example, in a setting such as a household, school or work setting with one SARS-CoV-2-positive individual, the other individuals in that setting will be probably more inclined to test themselves. Thereby, they could also detect possible (past) infections in sources and other contacts, especially when testing is widely available and media attention makes people aware. This could be different in circumstances where testing capacities and awareness are limited.

Fourthly, our study completely relied on reliability of self-report, which may be unreliable or subject to recall bias. Modelling studies may underestimate the impact of those factors.

Our findings differ from the results of the study by Raymenants et al. [18]. The discrepancy could be attributed to the different study periods. During our investigation, a partial lockdown was in effect, with most contacts being within households. We hypothesised that within these households, individuals tended to test earlier since they were immediately aware that a household member was infected, which could explain the high percentage of sources already known to the PHS. In contrast, Raymenants et al. conducted their study after 10 March 2021, when societal restrictions were gradually relaxed and ex-household social contacts began to increase. This might have led to individuals being less aware of potential infections outside their immediate surroundings. Moreover, we did not approach possible source contacts who were asymptomatic, whereas Raymenants et al. extended their testing to both symptomatic and asymptomatic contacts. 

Our study has some limitations. We performed this study during a specific pandemic context when there were considerable lockdown restrictions, limited inter-personal contacts and a low R_0_ between 0.97 and 1.17 [20]. Similarly, the role of ‘overdispersion’ and ‘superspreaders’, a crucial requirement to make BCT effective, might have been limited under these circumstances. Therefore, we cannot generalise our conclusions regarding BCT to all scenarios that could occur in the pandemic. In addition, we used traditional manual contact tracing as digital methods for BCT were not available. Digital contact tracing, where tracking systems on mobile phones, for example, can instantly identify and alert close contacts, could increase the number of sources identified and improve the timeliness of the contact tracing process. This is especially relevant considering the time-consuming process of manual contact tracing. However, such systems may only be effective if the coverage among a population is high, and they must be balanced with important ethical and legal considerations, notably around data privacy [21].

In our study, we found no COVID-19 cases in the first generation (G1), and therefore we could not evaluate the effects of tracing delays. Nevertheless, we hypothesise that it may play a more important role when BCT via asymptomatic cases can be investigated. To increase the effect of contact tracing for COVID-19, for example, when new variants of concern appear, forward contact tracing could be optimised by adding additional (digital) sources of information for tracing to allow quarantining contacts at risk.

## Conclusion

In conclusion, we found no benefit of supplementing forward contact tracing with our implementation of manual BCT for COVID-19 in practice during a partial lockdown in a population setting. We considered it a too laborious technique for the contact tracers and cumbersome for the extra contacts contacted.

## Supplementary Data

792000 Supplement
