# Supplementary material for: The value of manual backward contact tracing to control COVID-19 in practice, the Netherlands, February to March 2021: a pilot study
Source: Euro Surveill. 2023 Oct 12;28(41):2200916. doi: 10.2807/1560-7917.ES.2023.28.41.2200916 (PMC10571494; doi:10.2807/1560-7917.ES.2023.28.41.2200916)
Supplement: Supplement [file 22-00916_BOELSUMS_SUPPLEMENT.pdf]

Supplement

This supplementary material is hosted by Eurosurveillance as supporting information alongside the article 'The value of manual backward contact tracing to control COVID-19 in practice, the Netherlands, February to March 2021: a pilot study' on behalf of the authors who remain responsible for the accuracy and appropriateness of the content. The same standards for ethics, copyright, attributions and permissions as for the article apply. Eurosurveillance is not responsible for the maintenance of any links or email addresses provided therein.

Contents

- Supplementary figure **S1**, steps backward contact tracing
- Supplementary material **S2**, study protocol (in Dutch)
- Supplementary table **S3**, Outcomes of backward contact tracing of SARS-CoV-2 positive index cases by public health service Rotterdam-Rijnmond, February and March 2021
- Supplementary material **S4**, questionnaires backward contact tracing
- Supplementary figure **S5**, example backward contact tracing

Supplementary figure **S1**, steps backward contact tracing

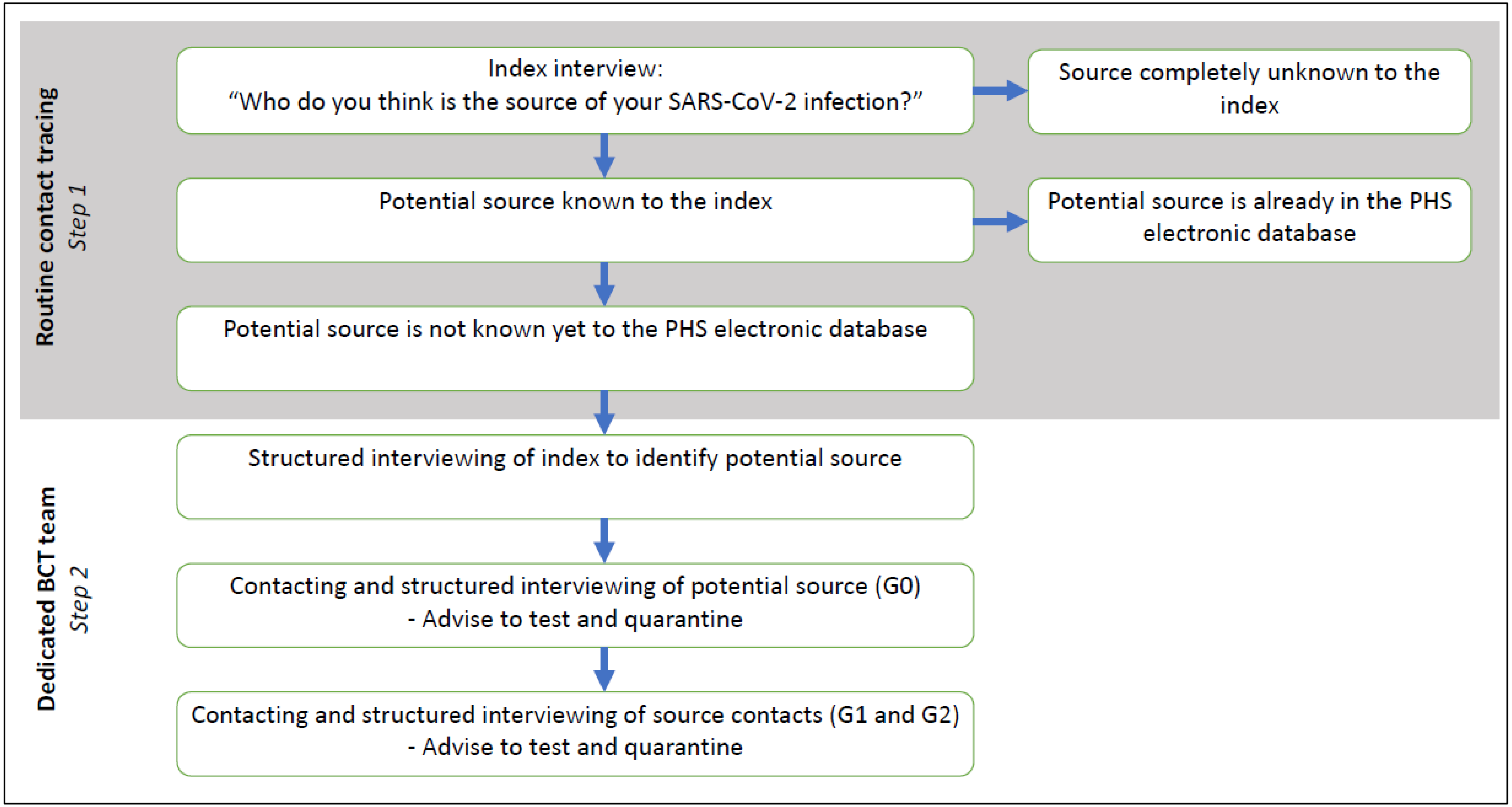

Supplementary material **S2**, study protocol (in Dutch)

GGD
Rotterdam-Rijnmond

Pilot

TEN TIJDE VAN **COVID-19**
PANDEMIE

Boelsums T.L. (Timo)

INHOUD

Pilot ... 3

Inleiding ... 6

Pilot ... 6

Rationale ... 7

Lijst met afkortingen ... 8

Werkinstructie ... 10

Werkverdeling ... 10

Werkwijze ... 10

Algemene informatie voor deelnemers ... 10

STAP 1. Regulier BCO ... 11

1.1 BCO uitvoeren ... 11

1.2 Bron uitvragen ... 11

Welke casussen zijn NIET geschikt voor backward tracing? ... 11

Welke casussen zijn WEL geschikt voor backward tracing? ... 11

1.3 Toestemming vragen & informeren ... 12

STAP 2. Vermoedelijke bron identificeren ... 13

2.1 Tijdlijn ... 13

2.2 Informatie verzamelen ... 13

2.3 Administratie ... 15

2.4 Vervolgstappen fase 2 ... 15

Risicolocaties ... 15

Brief sturen ... 16

STAP 3. Vermoedelijke ongeïdentificeerde bron (VOB) benaderen ... 17

3.1 Toestemming vragen & informeren ... 17

3.2 Informatie verzamelen ... 17

3.3. Broncontacten inventariseren ... 17

VOB heeft symptomen (gehad) ... 17

VOB heeft geen symptomen (gehad) ... 18

3.4 Locaties inventariseren ... 18

3.5 Dossier aanmaken 18
3.6 Test inplannen 18
3.7 Adviezen geven 19
Isolatie 19
Contacten 19
3.8 Vervolgstappen 19
Contacten 19
Locaties 19
Brief sturen 20
STAP 4. Broncontacten (BC) benaderen 21
4.1 Toestemming vragen & informeren 21
4.2 Informatie verzamelen 21
4.3 Contacten van broncontacten (CBC) inventariseren 21
Broncontact heeft symptomen (gehad) 21
Broncontact heeft geen symptomen (gehad) 22
4.4 Contactdossier aanmaken 22
4.5 Test inplannen & actie aanmaken 22
4.6 Vervolgacties 22
Isolatieadviezen BC 22
Contacten 22
Brief sturen 23
STAP 5. Contacten van Broncontacten (CBC) 24
5.1 Informatie verzamelen 24
5.2 Test inplannen 24
5.3 Informeren en Adviezen geven 24
Quarantaineadviezen 24
Contact GGD 25
Brief sturen 25
STAP 6. Follow-up 26
Contact CBC n.a.v. testuitslag BC 26

TIPS & TRICKS 27

Administratie 28

HP Zone 28

Bijlagen 29

Training 29

Stroomdiagrammen 29

Format 1: Indexgesprek 29

Format 2: Bronopsporing 32

Format 3: Vermoedelijke bron (VOB) 35

Format 4: Broncontact 36

Brondossier aanmaken 38

Doorverbinden afsprakenlijn 38

In de wacht zetten 38

Doorverbinden 38

Contactdossier aanmaken 39

Evaluatieformulier BCO-medewerker 41

Informatiebrieven ..... **Fout! Bladwijzer niet gedefinieerd.**

INLEIDING

Al sinds het begin van de COVID 19-pandemie zijn er aanwijzingen dat het virus zich onevenredig verspreidt in de populatie (overdispersion). Grote uitbraken (super spreading) kunnen ontstaan door enkele zeer besmettelijke personen. Andere patiënten lijken maar een beperkt aantal of geen nieuwe besmettingen te veroorzaken. Een gerichtere aanpak op deze super spreading events vanuit het bron- en contactonderzoek (BCO) levert mogelijk extra winst op bij het indammen van het virus.

Eerder zijn grote lokale uitbraken succesvol gedoofd door het herleiden van een hoog-besmettelijke bron en het opsporen en instrueren van zijn/haar contacten. Daarnaast tonen simulaties aan dat backward contact tracing (BWT) mogelijk effectiever is dan forward tracing bij het bron- en contactonderzoek (Endo 2020, Kojaku 2020). Op dit moment is backward contact tracing als onderdeel van het BCO nog niet expliciet uitgewerkt binnen de richtlijnen van de LCI.

PILOT

In een pilot zal bij een deel van de BCO's onderzocht worden wat de aanvullende waarde is van backward tracing bij het bron- en contactonderzoek van de GGD. Hierbij zal gekeken worden of het nieuwe cases en contacten vindt die niet in beeld waren gekomen bij het reguliere BCO.

Er zal worden gekeken naar de volgende **uitkomstmaten**:

- Wat is de extra werklast voor de BCO-medewerkers (extra tijd per uitgebreid brononderzoek)?
- Hoe bereid zijn de contacten om mee te werken (tijdsinvestering) en eventuele quarantainemaatregelen na te leven?
- Hoe haalbaar is het voor de contacten om betrouwbare informatie op te halen over gebeurtenissen (nauwe contacten, bezoek van locaties) in het verleden?
- Wat levert het brononderzoek op in aantal nog niet geïdentificeerde contacten en aantal voorkomen besmettelijke dagen?

RATIONALE

Het doel van backward tracing is het opsporen van vermoedelijk ongeïdentificeerde bronnen (VOB) en diens (mogelijk) blootgestelde contacten.

Vermoedelijk ongeïdentificeerde bron (VOB)

Momenteel wordt binnen het BCO met name gefocust op het contactonderzoek (*forward tracing*). Er valt mogelijk veel winst te behalen door opsporing een bron die eerder niet in beeld is gekomen door het ontbreken van klachten of die zich niet heeft laten testen om een andere reden.

In onderstaand voorbeeld zijn via index de contacten van index (IC) geïnventariseerd en in quarantaine geplaatst. Doordat de bron van index onbekend is, zijn de andere nauwe contacten van de bron (broncontacten = BC) niet op de hoogte van het feit dat zij in contact zijn geweest met iemand die het coronavirus bij zich draagt. Hierdoor zijn zij initieel al minder oplettend en zullen zij mogelijk bij (milde) klachten minder snel geneigd zijn zich ook te laten testen.

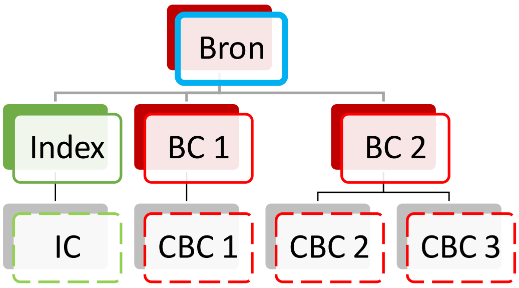

BC = Broncontact; CBC = Contact van broncontact; IC = Contact van index.

Besmettelijke dagen

Het doel is om de broncontacten op te sporen, te informeren en te testen. Gelijktijdig worden de contacten van de broncontacten (CBC) die nog het risico lopen om besmet te worden geïnformeerd over het verhoogde risico op besmetting en geadviseerd om in quarantaine te gaan totdat de testuitslag van de BC bekend is. Op deze manier wordt voorkómen dat blootgestelde CBC's op hun beurt andere nauwe contacten besmetten. Zie onderstaand voorbeeld.

Dag 8 (zie pijl) is het moment waarop de index een positieve testuitslag krijgt en het indexgesprek wordt gevoerd. Dit is het moment dat het brononderzoek opgestart kan worden. Bron besmet BC op dag 2. BC is niet op de hoogte van besmetting (niet geïdentificeerd als nauw contact en/of niet geïnformeerd) of niet in quarantaine. Op dag 7 krijgt BC klachten (uitgaand van een gemiddelde

incubatietijd van 5 dagen). Op dag 8 krijgt BC een test en op dag 9 informeert hij de CBC over de positieve uitslag. Op dag 5 en 6 heeft BC, mits hij direct in isolatie is gegaan na ontstaan van klachten, potentieel anderen kunnen besmetten. Wanneer BC op dag 5 nauw contact heeft gehad met CBC en dit contact hierbij heeft besmet, kunnen er klachten ontstaan op dag 10 (incubatietijd van 5 dagen). Besmettelijke periode van CBC is vanaf dag 6. Indien op dag 8 reeds de CBC worden geïnformeerd en in quarantaine gaan, voorkomt dit dat de CBC in hun besmettelijke periode anderen verder besmetten. In dit voorbeeld levert backward tracing – het identificeren van de bron en diens contacten – levert dus een 'winst' op van twee besmettelijke dagen per CBC dat in quarantaine gaat.

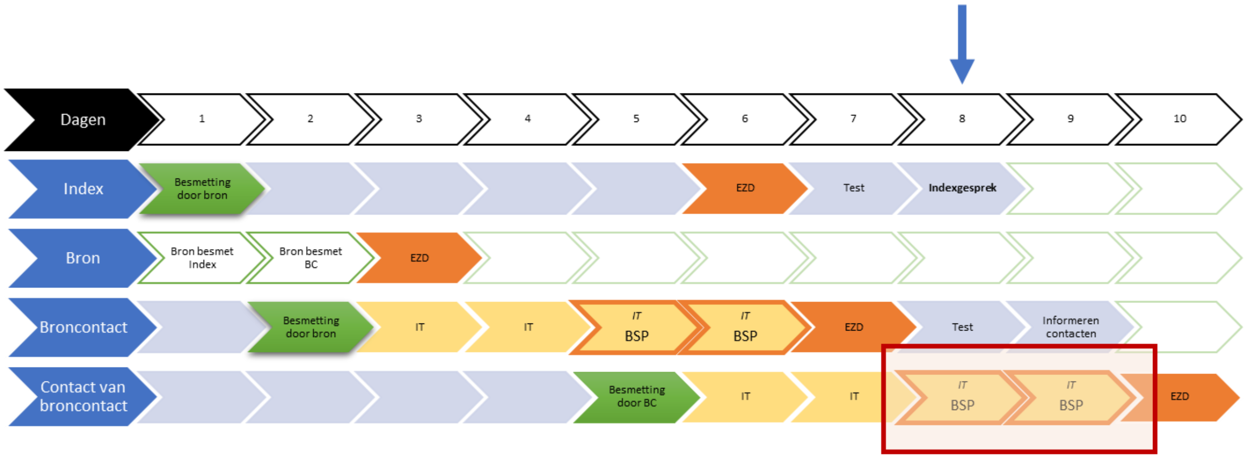

BC = Broncontact; BSP = besmettelijke periode; IT = incubatietijd; EZD = eerste ziektedag.

LIJST MET AFKORTINGEN

**Afkorting**	**Betekenis**
BC	Broncontact
BCO	Bron- en contactonderzoek
BSP	Besmettelijke periode
BWT	Backward (contact) tracing
CBC	Contact van broncontact
EZD	Eerste ziektedag
IC	Contact van index

IT	Incubatietijd
VOB	Vermoedelijke ongeïdentificeerde bron

WERKINSTRUCTIE

Op donderdag 03-12-20 is de pilot van start gegaan. In afstemming met de floormanagers en planning zal een groep van 12-15 (ervaren) BCO-medewerkers worden geselecteerd. Van tevoren ontvangen zij de werkinstructie die zij dienen door te nemen voor de start van de pilot. De belangrijkste informatie zal op de ochtend zelf nog kort worden gepresenteerd (zie bijlage '<u>Training</u>' voor een link naar de presentatie). Een geschikte ruimte zal worden geregeld in het Timmerhuis waar de groep gezamenlijk kan werken. Voor hen dient een Vraagbaak beschikbaar te zijn voor het deel 'regulier BCO'. De pilotcoördinator zal de gehele dag aanwezig zijn voor het beantwoorden van vragen.

WERKVERDELING

- BCO-medewerkers.
  - Telefonisch contact opnemen met index, VOB en diens contacten.
  - Registratie: formats invullen en (contact)dossiers aanmaken.
  - Tijdlijn/overzicht maken: tekenen of met format(s) uit deze werkinstructie.
  - Informeren contacten: pilot en beleid.
  - Evaluatie pilot(resultaten).
- Floormanagers en planning: BCO-medewerkers selecteren, planning maken, ruimte regelen.
- Pilotcoördinator: coördinatie pilot, briefing, beantwoorden van vragen en analyseren resultaten.

WERKWIJZE

Zie voor een overzicht van de werkinstructie m.b.v. stroomdiagrammen de bijlage '<u>Stroomdiagrammen</u>'.

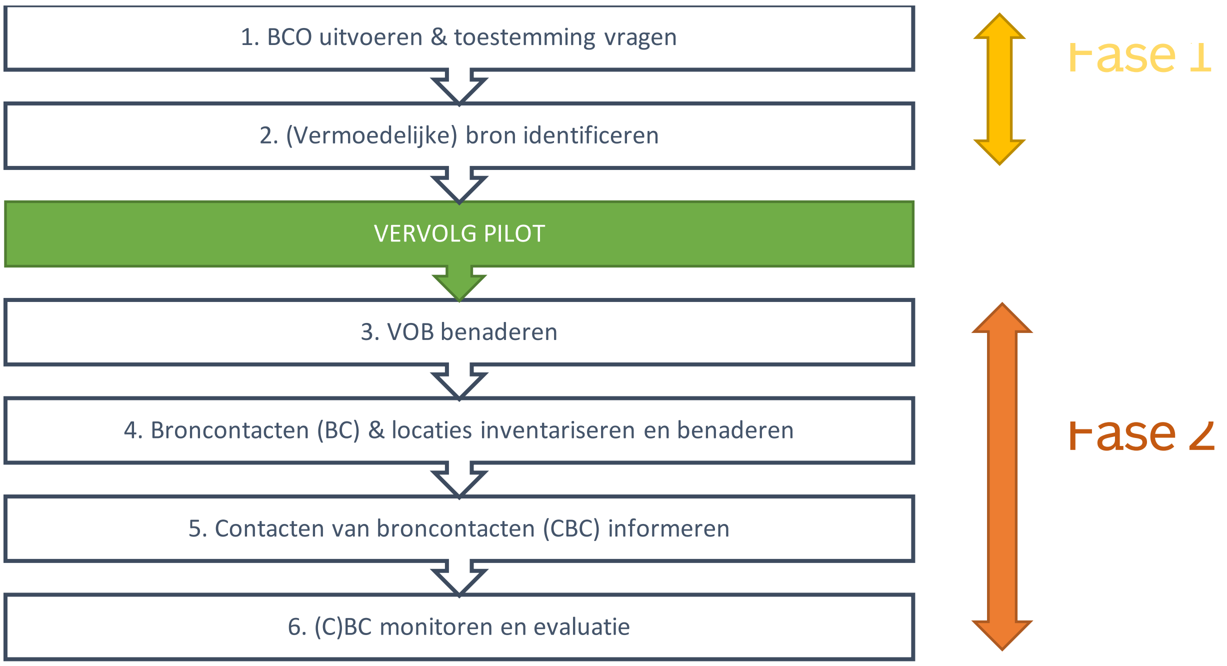

ALGEMENE INFORMATIE VOOR DEELNEMERS

Voor ieder soort deelnemer (index, VOB, BC en CBC) zijn apart <u>informatiebrieven</u> opgesteld. Lees deze goed door en informeer de deelnemers.

STAP 1. REGULIER BCO

1.1 BCO UITVOEREN

1. **Voer BCO volgens de reguliere werkinstructie uit.**
   Besteed nog geen extra aandacht aan het uitvragen van de bron.

2. **Vermeld in de Key details van het indexdossier**: PILOT BACKWARD TRACING.

3. **Maak een actie aan in het indexdossier, zodat de resultaten van de pilot makkelijk teruggevonden kunnen worden.**

a. Create New Action: Pilot BWT – index.
b. Assign to individual: Charlotte Hoffman.
c. Due date: today.
d. Vermeld in de Notes (vul deze eventueel aan na het BCO):

Bron bekend: [Ja / Nee]
Vermoedelijke bron: [Ja / Nee / Niet van toepassing]
Aantal VOB: [aantal] / Niet van toepassing
Aantal risicolocaties: [aantal] / Niet van toepassing

1.2 BRON UITVRAGEN

WELKE CASUSSEN ZIJN NIET GESCHIKT VOOR BACKWARD TRACING?

- Bekende of vermoedelijke bronnen met een positieve test: bij hen is reeds BCO uitgevoerd.

Voeg deze case dan (indien mogelijk) toe als *linked case* volgens de werkinstructie en rond het BCO verder af. Stop dan met de pilot, vul het aangepaste format 'Indexgesprek' in en plak het in een Event in het indexdossier.

- Bekende of vermoedelijke bron is <12 jaar oud.
- Asymptomatische huisgenoot.

LET OP: neem niet zomaar genoegen met de bron die index aanwijst. Ga na of het logisch is dat dit ook de daadwerkelijke bron is.
Bijvoorbeeld: index wijst vermoedelijke bron aan met wie index één dag voor EZD in contact is geweest. Het is onwaarschijnlijk dat de incubatieperiode slechts 1 dag bedraagt. Vermoedelijk is er dus een meer waarschijnlijke bron van de index.

WELKE CASUSSEN ZIJN WEL GESCHIKT VOOR BACKWARD TRACING?

- Bekende of vermoedelijke bron waarvan index weet dat deze niet is getest.
- Bekende of vermoedelijke bron waarvan index niet (zeker) weet of deze is getest.
- Onbekende bron.

Als de casus geschikt is, ga dan door met de volgende stap. Is de casus niet geschikt? Stop dan met de pilot, vul het aangepaste format 'Indexgesprek' in en plak het in een Event in het indexdossier. Het format 'Bronopsporing' hoeft dan niet ingevuld te worden.

1.3 TOESTEMMING VRAGEN & INFORMEREN

1. Vraag na het afronden van het reguliere BCO-gesprek of de index bereid is om mee te werken aan een nieuwe pilot van de GGD Rotterdam-Rijnmond.
2. Informeer de index over de pilot aan de hand van de informatie in onderstaand kader, leg uit waarom het belangrijk is en vraag of hij/zij wil meewerken. Benadruk dat deelname hieraan geheel vrijwillig is.
3. Wil index niet meewerken? Stop dan met de pilot, vul het aangepaste format 'Indexgesprek' in en plak het in een Event in het indexdossier. Ga anders naar stap 2.

Informatie voor BCO

- Nieuw onderzoek op kleine schaal naar de meerwaarde van intensieve bronopsporing
- Is arbeidsintensief en wordt daarom op dit moment nog niet (uitgebreid) uitgevoerd
- De pilot is verdeeld in 2 fases:
  - Fase 1 pilot is gericht op een uitgebreidere bronopsporing.
  - Fase 2 is gericht op het contact opnemen met de vermoedelijke bronnen en diens contacten.
- Ga pas door met fase 2 na overleg met de pilotcoördinator waarbij de vermoedelijke bronnen (en diens contacten) worden gecontacteerd.

Wat betekent dit voor index?

- Tijdsinvestering: uitgebreid nagaan van de dagen waarop index mogelijk besmet kan zijn geraakt.
- Het kan zijn dat het contact geschikt is voor fase 2, waarbij wij (na toestemming) contact zullen opnemen met dit contact.
- Bijdragen aan belangrijk nieuw onderzoek om virusverspreiding verder in te dammen.
- Index wordt eventueel teruggebeld voor toestemming om contact op te nemen met vermoedelijke bron(nen).

STAP 2. VERMOEDELIJKE BRON IDENTIFICEREN

Als niet duidelijk wie de bron van index is of waar index (mogelijk) besmet is, voer dan de volgende stappen uit.

2.1 TIJDLIJN

1. **Bepaal in welke periode index het meest waarschijnlijk is besmet.**
   Neem hiervoor de periode van 3 dagen voor EZD tot 8 dagen voor EZD.
   Bijvoorbeeld: EZD van index is op 10 november. De periode waarin index het meest waarschijnlijk is besmet is van 2 november (= 8 dagen voor EZD) t/m 7 november (= 3 dagen voor EZD).

2. **Maak voor jezelf een tijdlijn.**
   Tip: voor een handige tijdlijn, gebruik het Excelformat in de map Werkinstructie.

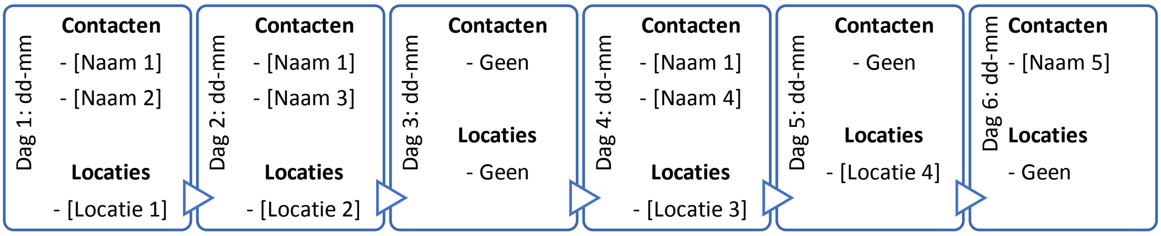

3. **Vraag per dag in deze periode goed en nauwkeurig uit met wie en waar index is geweest.**
- Benoem de mogelijk personen die in die periode langs is/zijn geweest: kinderen, ouders, buren, familie, vrienden, monteur, huishoudelijke hulp, koerier, etc.
- Geef de index desnoods tijd om hierover na te denken en bel eventueel op een later tijdstip terug.

4. **Vraag kort na of er buiten deze periode van -8 tot -3 voor EZD een vermoedelijke bron ligt.**

5. **Geen contacten gehad of locaties bezocht in deze periode?**
   Stop de pilot en rond het BCO verder af zoals gebruikelijk. Wel contacten gehad? Ga dan door naar stap 2.2.

2.2 INFORMATIE VERZAMELEN

1. **Maak per contact een risicoschatting of het contact de vermoedelijke ongeïdentificeerde bron (VOB) van index kan zijn aan de hand van onderstaande vragen.**

Risicoschatting contacten

- Is het contact een nauw contact?
  Was het contactmoment >15 min. op <1.5 meter afstand?
- Is het een overig contact dat kan worden beschouwd als risicocontact?
  - Had het contact (milde) symptomen tijdens het contactmoment of heeft hij/zij nadien alsnog klachten ontwikkeld?
  - Is het onduidelijk of het contact tijdens het contactmoment symptomen had of klachten heeft gekregen nadien?
  - Is er een andere reden waarom kan worden aangenomen dat dit contact de vermoedelijke bron kan zijn, bijvoorbeeld:
    - *Risico contactmoment (hoesten, zoenen, etc.);*
    - *Het is het enige contact dat index heeft gezien;*
    - *Er is sprake geweest van frequent en intensief contact tussen index en bron;*
    - *Bron heeft zelf in omgeving mensen met klachten en/of positieve*

2. **Verzamel informatie over het contactmoment voor ieder nauw én risicocontact.**

- Naam en contactgegevens.
  Let op: wees voorzichtig met het direct stellen van deze vraag! Beter kan eerst een beeld worden verkregen van de situatie alvorens je deze gevoelige gegevens uitvraagt.
- Datum/data?
- Aard van het contact (tijdsduur, afstand, risicocontactmoment)?
- Relatie tot index (vriend, huisgenoot, docent, etc.)?
- Locatie (binnen/buiten, grootte, ventilatie, etc.)?
- Risicocontact (contact had klachten, hoog-risico contact (hoesten/niezen/zoenen), anderen in omgeving met klachten en/of positieve test)?
- Geeft index toestemming om contact op te nemen met dit contact? Geeft index toestemming om naam te noemen?

3. **Ga per locatie af of hier mogelijk de bron van de besmetting heeft plaatsgevonden.**

Risicoschatting locaties

- Is index ergens geweest waar anderen aanwezig waren die nadien (ongeveer rond dezelfde tijd) ook klachten hebben ontwikkeld?
- Is het een locatie waar er grote kans is op transmissie?
  Bijvoorbeeld kleine, drukbezochte en slecht geventileerde ruimtes waar moeilijk afstand gehouden kan worden.
- Zijn er aanwijzingen dan index onderdeel is van een cluster/situatie/uitbraak?

4. **Verzamel informatie over het contactmoment:**

- Wat is de naam van de locatie?
- Datum/data van bezoek?
- Soort locatie?
- Geef een omschrijving van de omstandigheden (ruimte, grootte, aantal mensen, ventilatie, etc.)

5. **Rond na uitvoeren van bovenstaande het gesprek met de index af en bedenk hem/haar voor de extra moeite en tijd die hij/zij heeft willen leveren voor een bijdrage aan dit onderzoek.**

2.3 ADMINISTRATIE

6. **Vul het aangepaste format 'Indexgesprek' in en plak het in een Event in het indexdossier.**

7. **Vul het format 'Bronopsporing' in en plak het in een apart Event.**
Indien meerdere bron(locaties): maak een volgorde van meest waarschijnlijke naar minst waarschijnlijke vermoedelijke bronnen. Op deze manier kunnen we bepalen welke VOB de meeste prioriteit hebben om eventueel te benaderen. Dit hoeft niet als de casus niet geschikt was voor de pilot (en dit dus niet is uitgevraagd).

8. **Bespreek met de pilotcoördinator de uitkomst van het pilotgesprek.**
LET OP: *Voordat er eventueel contact mag worden opgenomen met VOB moet dit eerst zijn overlegd met de pilotcoördinator.*

2.4 VERVOLGSTAPPEN FASE 2

Is in overleg met de pilotcoördinator besloten dat er contact mag worden opgenomen met de VOB? Ga dan verder met de volgende stappen.

1. **Bel na overleg met de pilotcoördinator (alle) VOB(s) uit de incubatieperiode. Vraag per contact uit:**
   - Heeft dit contact klachten (gehad)? Zo ja, wat voor klachten en in welke periode?
   - Heeft dit contact mensen in de omgeving met klachten verdacht voor COVID en/of een positieve test?
   - Wil dit contact meewerken aan de pilot en mag hij/zij op een later moment hiervoor teruggebeld worden?

2. **Bepaal in overleg met de pilotcoördinator of het contact kan worden beschouwd als vermoedelijke bron om verder te kunnen gaan naar fase 2.**
Vermeld de uitkomst in het format Bronopsporing.

3. **Ga door met stap 3 van deze werkinstructie als VOB geschikt is voor fase 2 van backward tracing.**

RISICOLOCATIES

BESPREEK MET PILOTCOÖRDINATOR IN HOEVERRE DEZE STAP GEWENST IS.

Context bestaat

1. Koppel context.
2. Is één van de volgende criteria van toepassing?
   a. ≥1 andere case die hier mogelijk is besmet (d.w.z. EZD ligt NA bezoek aan deze locatie) is op dezelfde dag in deze context geweest EN heeft hier (mogelijk) nauw contact gehad met index;
   b. Is één van de factoren uit kader *Risicoschatting locaties* van toepassing?
3. Overleg met pilotcoördinator of contact moet worden opgenomen met deze locatie. Anders geen verdere actie.

Context bestaat nog niet

- Vraag context aan op gebruikelijke wijze.
- Is één van de factoren uit kader *Risicoschatting locaties* van toepassing?
- Overleg met pilotcoördinator of contact moet worden opgenomen met deze locatie. Anders geen verdere actie.

BRIEF STUREN

Verwerk de gegeven adviezen en referentienummer (HP-nummer van gelinkt dossier) in de brief voor de index en stuur deze op. Bedank de index voor deelname aan de pilot.

STAP 3. VERMOEDELIJKE ONGEÏDENTIFICEERDE BRON (VOB) BENADEREN

3.1 TOESTEMMING VRAGEN & INFORMEREN

1. Vraag of de VOB bereid is om mee te werken aan een nieuwe pilot van de GGD Rotterdam-Rijnmond.
   LET OP: deelname is geheel vrijwillig. Deelnemers kunnen niet worden verplicht om zich te laten testen of de quarantaineadviezen op te volgen.
2. Informeer de VOB aan de hand van de juiste informatiebrief.
3. Vraag of de VOB toestemming geeft om contact op te nemen met zijn/haar contacten en zijn/haar naam te noemen om zo de volgende stap van het proces te kunnen uitvoeren.
4. Indien VOB geen toestemming geeft om (ook niet anoniem) contact op te nemen met diens contacten **stop pilot**. Ga anders door naar de volgende stap.

3.2 INFORMATIE VERZAMELEN

1. Vul deel 1 van het format 'Vermoedelijke bron' aan (bijv. contactgegevens van VOB die index niet had).
2. Vul deel 2 van het format 'Vermoedelijke bron' in aan de hand van onderstaande vragen.
3. Heeft de VOB zich inmiddels al getest en is er al BCO uitgevoerd? Bedank de VOB dan voor de medewerking en stop de pilot voor deze VOB.

Heeft VOB klachten (gehad)?*

- Eerste ziektedag (EZD).
- Beloop van symptomen.
- Indien hersteld: hersteldatum?
- Restklachten?

Heeft VOB andere contacten die ziek zijn geworden of positief zijn getest in de afgelopen 2 weken?

- Wat is de datum van het contactmoment?
- Geef een zo nauwkeurig mogelijke omschrijving van het contactmoment.
- Wanneer is/zijn deze perso(o)n(en) ziek geworden en/of positief getest?

Is VOB recent getest of heeft hij/zij een test ingepland?

- Wat is/was de datum (en evt. uitslag) van de test?
- Indien wel klachten en nooit laten testen: reden waarom niet laten testen?

* **LET OP**: vraag ook altijd goed door naar eventuele milde klachten (zoals neusverkoudheid, reuk- of smaakverlies) of atypische klachten (alles dat veranderd is ten opzichte van 'normale' staat van zijn).

3.3. BRONCONTACTEN INVENTARISEREN

Inventariseer de **nauwe contacten van de bron** (= broncontacten). De periode waarvan de nauwe contacten moeten worden geïnventariseerd is afhankelijk van of de VOB symptomen heeft of niet.

VOB HEEFT SYMPTOMEN (GEHAD)

1. Inventariseer de broncontacten in *de besmettelijke periode*.
   - Start besmettelijke periode: vanaf twee dagen voor start van symptomen.
   - Eind van besmettelijke periode:
     - Tot start isolatie; OF
     - Als VOB 24 uur klachtenvrij is én >7 dagen na EZD.

2. Vermeld per nauw contact: naam, contactgegevens en korte omschrijving van het contactmoment.
3. Vul deel 3 van het format 'Vermoedelijke bron' in.

VOB HEEFT GEEN SYMPTOMEN (GEHAD)

1. Inventariseer de broncontacten in *de periode in periode +3 tot -3 dagen tussen contactmoment van index en VOB*.
   Bijvoorbeeld: contactmoment tussen index en VOB is op 10 november. De periode waarvan de broncontacten moeten worden geïdentificeerd zijn van 7 november t/m 13 november.
2. Inventariseer per nauw contact: naam, contactgegevens en korte omschrijving van het contactmoment.
3. Vul deze gegevens in bij deel 3 van het format 'Vermoedelijke bron'.

3.4 LOCATIES INVENTARISEREN

1. Inventariseer iedere locatie waar de VOB is geweest in zijn/haar *besmettelijke periode*.
2. Ga per locatie af of de VOB hier mogelijk anderen heeft besmet aan de hand van de onderstaande criteria.

Risicoschatting locaties

- Is VOB ergens geweest waar anderen aanwezig waren die nadien (ongeveer rond dezelfde tijd) ook klachten hebben ontwikkeld?
- Is het een locatie waar er grote kans is op transmissie?
  Bijvoorbeeld kleine, drukbezochte en slecht geventileerde ruimtes waar moeilijk afstand gehouden kan worden.
- Zijn er aanwijzingen dan index onderdeel is van een cluster/situatie/uitbraak?

3. Vormt (één van de) locatie(s) een mogelijke risicolocatie? Vul de naam, adres en omschrijving van het contactmoment in bij *Locaties* van deel 3 van het format 'Vermoedelijke bron'.

3.5 DOSSIER AANMAKEN

1. Maak een '*Probable case*' dossier aan (zie bijlage Brondossier aanmaken).
2. Vermeld in de Key details: PILOT BACKWARD TRACING.
   a. Create 'New Action': *Pilot BWT – VOB.*
   b. Assign to individual: Charlotte Hoffman.
   c. Due date: today.
3. Plak het format 'Vermoedelijke bron' in een Event.

3.6 TEST INPLANNEN

Bespreek met de VOB dat wij graag een test zouden inplannen voor de VOB, ook als hij/zij ondertussen al geen klachten meer heeft. Index is niet verplicht om dit te doen. Het kan zijn dat de VOB (inmiddels) negatief test, maar dit zegt niets over of hij/zij de bron is geweest. De testuitslag heeft (waarschijnlijk) ook weinig consequenties. Het is belangrijker dat de broncontacten worden getest, omdat dit mogelijk nog gevolgen kan hebben voor het voorkomen van verdere verspreiding.

Gaat de VOB akkoord met het inplannen van een test, verbind de deelnemer (aan het eind van het gesprek) 'warm' door met een medewerker van de Afsprakenlijn (010 – 443 8031); zij zijn op de hoogte van de pilot *Backward tracing*. Vergeet niet het HP-nummer te vermelden van de persoon zelf (VOB-dossier).

Voor een korte instructie over doorverbinden, zie Doorverbinden afsprakenlijn.

3.7 ADVIEZEN GEVEN

ISOLATIE

Verwerk de gegeven adviezen in deel 4 van het format Vermoedelijke bron,

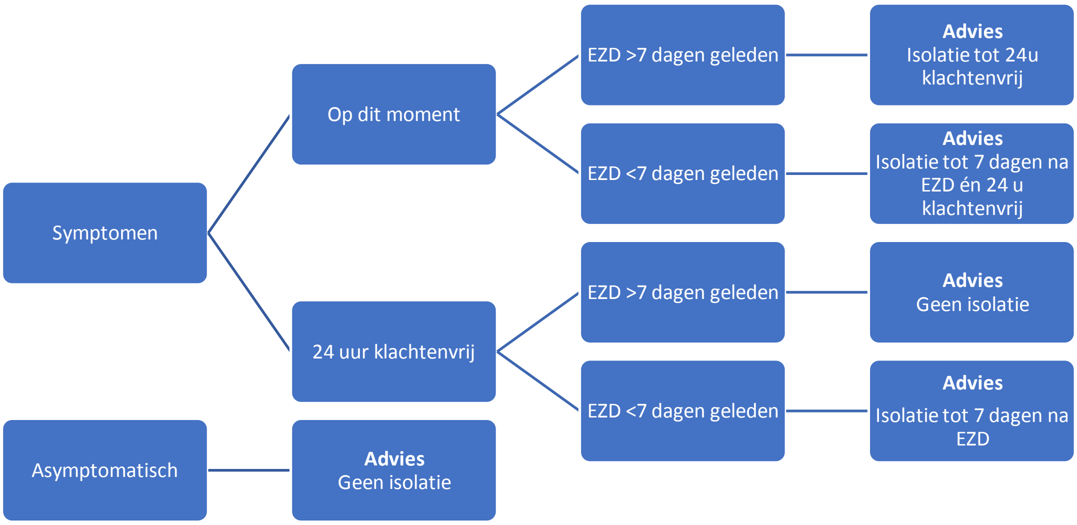

CONTACTEN

Wij nemen contact op met de nauwe contacten van de VOB en zullen hen adviseren om zich te laten testen en eventueel in quarantaine te gaan.

3.8 VERVOLGSTAPPEN

Heeft VOB geen nauwe of risicocontacten gehad of locaties bezocht in deze periode? Stop verder contactonderzoek voor deze VOB en rond het BCO verder af zoals gebruikelijk.

CONTACTEN

Voer voor ieder broncontact stap 4 uit.

LOCATIES

Bekijk voor iedere aangemerkte risicolocatie bij 'Locaties inventariseren' of de context bestaat.

Context bestaat

- Koppel context.
- Is één van de volgende criteria van toepassing?

    - ≥1 andere case die op dezelfde dag in deze context is geweest: deze case heeft hier (mogelijk) nauw contact gehad met index EN EZD van case ligt 2-14 dagen na bezoek aan deze locatie.
    - Locatie is aangemerkt als risicolocatie.
- Overleg met pilotcoördinator of contact moet worden opgenomen met deze locatie..

Context bestaat nog niet

- Vraag context aan op gebruikelijke wijze.
- Overleg met pilotcoördinator of arts of contact moet worden opgenomen met deze locatie.

BRIEF STUREN

Verwerk de gegeven adviezen en referentienummer (HP-nummer van gelinkt dossier) in de brief voor de VOB en stuur deze op. Bedank de VOB voor deelname aan de pilot.

STAP 4. BRONCONTACTEN (BC) BENADEREN

4.1 TOESTEMMING VRAGEN & INFORMEREN

1. Benader alle geïnventariseerde broncontacten.
   Vraag of de broncontact bereid is om mee te werken aan een nieuwe pilot van de GGD Rotterdam-Rijnmond.
   LET OP: deelname is geheel vrijwillig. Deelnemers kunnen niet worden verplicht om zich te laten testen of de quarantaineadviezen op te volgen als zij slechts (tweedegraads) contact zijn van een vermoedelijke bron.
2. Informeer de broncontact aan de hand van de juiste informatiebrief.
3. Vraag of de broncontact toestemming geeft om contact op te nemen met zijn/haar contacten en zijn/haar naam te noemen om zo de volgende stap van het proces te kunnen uitvoeren.
4. Vraag of broncontact bereid is om te testen.

Indien BC geen toestemming geeft om (ook niet anoniem) contact op te nemen met diens contacten EN/OF niet bereid is om te testen stop pilot voor deze BC. Ga anders door naar de volgende stap.

4.2 INFORMATIE VERZAMELEN

- Vraag naam, e-mailadres, BSN en/of geboortedatum uit om in een contactdossier aan te maken.
- Vul deel 1 van het format 'Broncontact' in aan de hand van onderstaande vragen.

Heeft BC klachten (gehad)?*

- Eerste ziektedag (EZD).
- Beloop van symptomen.
- Indien hersteld: hersteldatum?
- Restklachten?

Omschrijf het contactmoment tussen VOB en BC zo nauwkeurig mogelijk.
Is VOB recent getest of heeft hij/zij een test ingepland?

- Wat is/was de datum (en evt. uitslag) van de test?
- Indien wel klachten en nooit laten testen: reden waarom niet laten testen?

* **LET OP**: vraag ook altijd goed door naar eventuele milde klachten (zoals neusverkoudheid, reuk- of smaakverlies) of atypische klachten (alles dat veranderd is ten opzichte van 'normale' staat van zijn).

4.3 CONTACTEN VAN BRONCONTACTEN (CBC) INVENTARISEREN

Welke nauwe contacten van de BC (= CBC) moeten worden geïnventariseerd is afhankelijk van of BC klachten heeft (gehad).

BRONCONTACT HEEFT SYMPTOMEN (GEHAD)

1. Inventariseer de nauwe contacten van het broncontact (CBC) *in de besmettelijke periode*.
   - Start besmettelijke periode: vanaf twee dagen voor start van symptomen.
   - Eind van besmettelijke periode:
     - Tot start isolatie; OF
     - Als VOB 24 uur klachtenvrij is én >7 dagen na EZD.

2. Vermeld per CBC: naam, contactgegevens en korte omschrijving van het contactmoment.
3. Vul deel 2 van het format 'Broncontact' in.

BRONCONTACT HEEFT GEEN SYMPTOMEN (GEHAD)

Als BC geen klachten heeft (gehad), dient BC zelf zijn/haar contacten te informeren en hen te adviseren om voorzichtig te zijn met het bezoeken van risicogroepen en drukbezochte locaties totdat de testuitslag van BC bekend is.

4.4 CONTACTDOSSIER AANMAKEN

1. Maak voor ieder broncontact een contactdossier aan. Zie bijlage Contactdossier aanmaken.
2. Plak het format 'Broncontact' in een Event van het dossier.

4.5 TEST INPLANNEN & ACTIE AANMAKEN

1. Verbind de deelnemer (aan het eind van het gesprek) 'warm' door met een medewerker van de Afsprakenlijn (010 – 443 8031); zij zijn op de hoogte van de pilot *Backward tracing*. Vergeet niet het HP-nummer te vermelden van het dossier waaraan de deelnemer gelinkt is.
   NB: Voor een korte instructie over doorverbinden, zie Doorverbinden afsprakenlijn.

2. Maak in **ieder contactdossier** een actie aan.
   a. Create 'New Action': *Pilot BWT – Broncontact.*
   b. Assign to individual: Charlotte Hoffman.
   c. Due date: today.

3. Heeft broncontact symptomen (gehad) EN heeft hij/zij nauwe contacten opgegeven die (waarschijnlijk) in quarantaine gaan? Maak dan ook de volgende actie aan:
   a. Create 'New Action': *Pilot BWT – contact CBC.*
   b. Assign to individual: Charlotte Hoffman.
   c. Due date: vul hier de datum in *1 dag na (verwachte) testdatum van broncontact*. *Bijvoorbeeld: je hebt voor broncontact test ingepland op 10 november. Zet de due date dan op 11 november.*

4.6 VERVOLGACTIES

ISOLATIEADVIEZEN BC

Verwerk de gegeven adviezen in deel 1 van het format Broncontact.

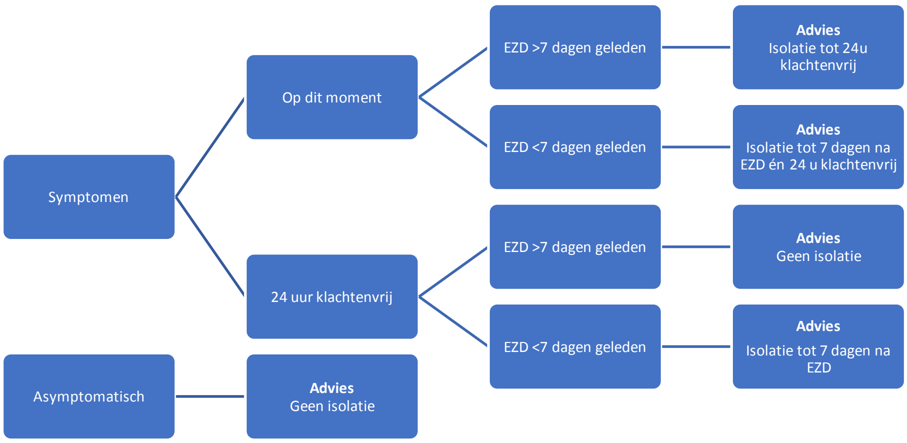

CONTACTEN

- BC heeft symptomen (gehad): wij nemen contact op met nauwe contacten van BC. Wij zullen hen informeren en adviseren om in quarantaine te gaan tot de testuitslag van BC bekend is.
    - BC testuitslag negatief: BC krijgt testuitslag op gebruikelijke wijze van de GGD. Wij zullen de nauwe contacten van BC (CBC) informeren.
    - BC testuitslag positief: BC wordt gebeld voor regulier BCO. De CBC worden benaderd als nauwe contacten van BC.
- BC heeft geen symptomen (gehad): BC dient zelf zijn/haar contacten te informeren en hen te adviseren om voorzichtig te zijn met het bezoeken van risicogroepen en drukbezochte locaties totdat de testuitslag van BC bekend is.

BRIEF STUREN

Verwerk de gegeven adviezen en referentienummer (HP-nummer) in de brief voor de BC stuur deze op. Bedank de BC voor deelname aan de pilot.

STAP 5. CONTACTEN VAN BRONCONTACTEN (CBC)

1. Benader alle geïnventariseerde CBC uit stap 4.
   Vraag of CBC bereid is om mee te werken aan een nieuwe pilot van de GGD Rotterdam-Rijnmond.
   LET OP: deelname is geheel vrijwillig. Deelnemers kunnen niet worden verplicht om zich te laten testen of de quarantaineadviezen op te volgen als zij slechts (tweedegraads) contact zijn van een vermoedelijke bron.
2. Informeer de CBC aan de hand van de juiste informatiebrief.

5.1 INFORMATIE VERZAMELEN

Vul deel 2 van het format Broncontact in aan de hand van de volgende vragen.

Naam en contactgegevens van CBC?
Heeft CBC klachten (gehad)?*

- Eerste ziektedag (EZD).
- Beloop van symptomen.
- Indien hersteld: hersteldatum?
- Restklachten?

Omschrijf het contactmoment tussen VOB en BC zo nauwkeurig mogelijk

5.2 TEST INPLANNEN

Voor CBC wordt alleen een test ingepland als hij/zij symptomen heeft. Als CBC geen klachten heeft (gehad) dan worden er alleen adviezen gegeven en een informatiebrief verstuurd.

Verbind de symptomatische CBC (aan het eind van het gesprek) ‘warm’ door met een medewerker van de Afsprakenlijn (010 – 443 8031); zij zijn op de hoogte van de pilot *Backward tracing*. Vergeet niet het HP-nummer te vermelden van het dossier waaraan de deelnemer gelinkt is.

Voor een korte instructie over doorverbinden, zie Doorverbinden afsprakenlijn.

5.3 INFORMEREN EN ADVIEZEN GEVEN

QUARANTAINEADVIEZEN

Verwerk de gegeven adviezen in deel 2 van het format Broncontact.

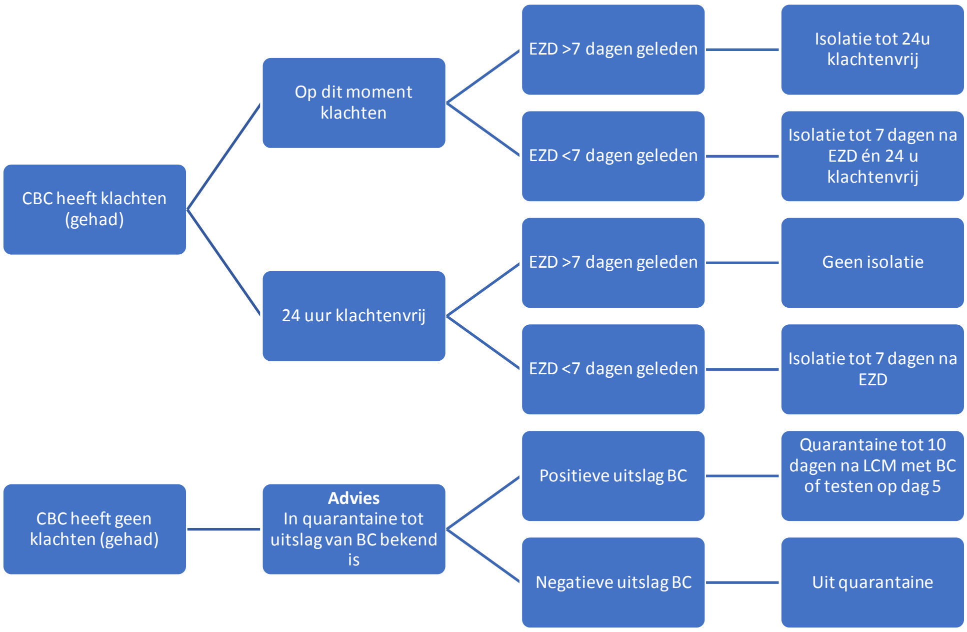

CONTACT GGD

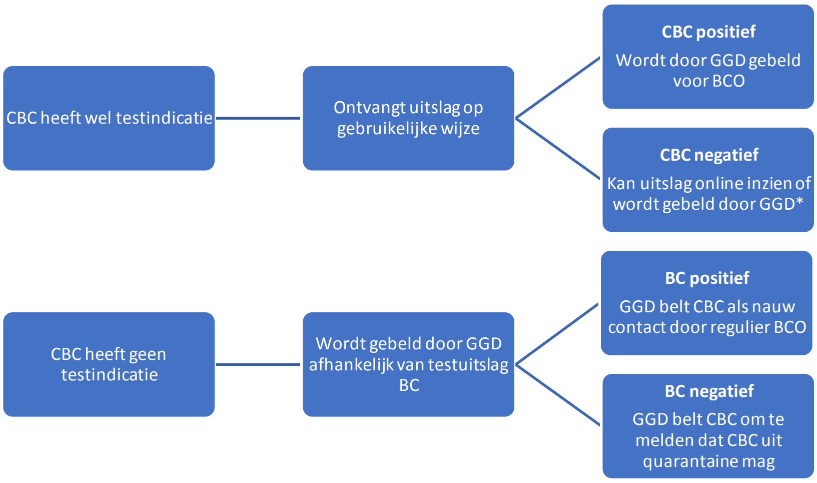

*Test BC positief EN is de test CBC <5 dagen na laatste contactmoment tussen BC en CBC? Adviseer dan om de quarantaine af te maken of een hertest te doen op dag 5.

BRIEF STUREN

Verwerk de gegeven adviezen en referentienummer (HP-nummer) in de brief voor de CBC en stuur deze op. Bedank de CBC voor deelname aan de pilot.

STAP 6. FOLLOW-UP

CONTACT CBC N.A.V. TESTUITSLAG BC

In de workload BCO Pilot staan alle dossiers die zijn verbonden aan deze pilot. De acties 'BWT – contact CBC' zijn van belang om de CBC te bellen in het geval van een negatieve testuitslag van BC.

TIPS & TRICKS

Naar aanleiding van de eerste dagen van de pilot, hebben we een aantal tips toegevoegd aan dit bestand aan de hand van de feedback.

- Het is belangrijk om ALLE contacten en locaties echt met iemand af te gaan.
  - Als een index zelf niet vermoedt dat iets een risicolocatie of contact is, zal hij/zij deze ook niet snel uit zichzelf noemen als dusdanig.
- Wees kritisch!
  - Een contact dat index zelf aanwijst als vermoedelijke bron, is niet altijd een contact wat wij ook zouden aanwijzen als VOB.
- Begin met de vraag wat iemand heeft gedaan in plaats van direct de vraag te stellen wie de contacten zijn.
- Tips voor gesprekstechnieken.
  - Open start: niet direct de VOB aanwijzen als bron, maar dit open laten ("U bent één van de contacten die we benaderen").
  - Aangeven dat je samen met VOB het netwerk wil 'ontrafelen': "U bent vast ook benieuwd wie de bron is van [naam index]..!"
  - VOB belonen: "We doen onderzoek naar de periode waarin [naam index] besmet is geraakt. Dat betekent dat we in dit onderzoek ook alle contacten van [naam index] uit deze periode benaderen en bij (milde) klachten (op dit moment of in de afgelopen weken) een test willen aanbieden. Als u zelf klachten heeft gehad, betekent dit ook dat wij al uw nauwe contacten in deze periode direct een test willen aanbieden. U helpt hierbij mee om de verspreiding in de kiem te smoren; uw bijdragen is zeer belangrijk."
- Duid je op weerstand?
  Explореer de reden van deze weerstand: "Waar bent u bang voor?"

ADMINISTRATIE

HP ZONE

Het BCO dient op gebruikelijke wijze te zijn afgerond en geadministreerd in HP Zone. Aan eind van het brononderzoek dienen de volgende gegevens te zijn vermeld in HP Zone:

- Vermeld in ieder dossier in de Key details (in contactdossier 'Additional comments' onder Key Details: PILOT BACKWARD TRACING.
  Op deze manier kunnen we de dossiers die zijn verbonden aan deze pilot makkelijk terugvinden.
- In ieder dossier dient een nieuwe actie te zijn aangemaakt voor de Workload BCO Pilot op naam van Charlotte Hoffman. Op deze manier kunnen de deelnemers aan de pilot makkelijk worden teruggevonden.
- Indexdossier: Format 'Indexgesprek' en 'Bronopsporing'.
- Brondossier: Format 'Vermoedelijke bron'.
- Contactdossier: Format 'Broncontact'.

BIJLAGEN

TRAINING

Link Powerpoint

STROOMDIAGRAMMEN

Link stroomdiagrammen

FORMAT 1: INDEXGESPREK

**Gesprek gevoerd met**	Index / [partner/kind/familielid/etc.] van index + [reden]
**Klinische gegevens**	
Datum testafname	
Reden van testen	
Eerste ziektedag (EZD)	
Klachten	[…] / asymptomatisch
Huidige klachten	[…] / hersteld, sinds […] / restklachten, [namelijk…]
Ziektes/aandoeningen (incl. overgewicht)	Nee / Ja, [naam aandoening]
Verzwakt afweersysteem door ziekte of medicatie?	Nee / Ja, [reden]
Zwangerschap of recent bevallen	Nee / N.v.t. / Ja, [aantal weken zwangerschap of datum van bevalling]
Naam huisarts	
**Prioritering**	
Huidige verblijfsplaats	Thuis / (zorg)instelling / daklozen- of asielzoekerscentrum / anders, [namelijk…]
Recente ziekenhuisopname	Nee / Ja, [naam ziekenhuis, afdeling, datum opname en evt. ontslag, IC-opname]
Betrokkenheid hulpdiensten (bijv. politie, ambulance)	Nee / Ja, [aard, duur, beschermd/onbeschermd]
Thuiszorg ontvangen	Nee / Ja, [type zorg] bij [naam instelling, plaats] op [datum, (on)beschermd?]

Zorginstelling bezocht (bijv. ziekenhuis, verzorgingshuis)	Nee / Ja, [naam instelling, plaats, afdeling] op [datum], aard van contacten
Bezoek of visite huisarts(enpost)	Nee / Ja, [naam, aard van bezoek, (on)beschermd]
Verblijf in buitenland of gevlogen/gebruik van groepsvervoer	Nee / Ja, [land(en) + data + vervoersmiddel en/of vluchtgegevens + accommodatie + evt. bijzonderheden tijdens verblijf]
Bezoeker/medewerker school of kinderdagverblijf	Nee / Ja, [naam organisatie, locatie, rol/functie], [wel/niet] in besmettelijke periode, [wel/geen] nauw contact
**Beroep**	[werkzaam / gepensioneerd / n.v.t.]
Organisatie, locatie, afdeling	
Functie	
Laatste werkdag	
Gewerkt tijdens besmettelijke periode	Nee / Ja, op [data]
Nauw contact op werk	Nee / Ja, met […] collega's, [toelichting]
Aantal overige contacten	
Collega's met klachten	Nee / Ja, [namelijk…]
Maatregelen op de werkvloer	Nee / Ja, [namelijk…]
Onrust op werk	Nee / Ja, [toelichting]
Contactpersoon/-gegevens organisatie	[Naam], [contactgegevens]
Werkgever reeds op de hoogte	Ja / Nee, index gaat werkgever zelf [wel/niet] op de hoogte stellen
**Brononderzoek**	
Bron	Bekend [incl. HP-nummer] / Vermoedelijk [incl. HP-nummer] / Onbekend
Bronopsporing (indien bron onbekend)	

Incubatieperiode<br><br>Nauwe contacten of risicocontacten in deze periode<br><br>Risicolocatie<br><br>Mogelijk onderdeel van cluster/situatie/uitbraak?	[datum] – [datum]<br><br>Ja, [aantal] / nee<br><br>Ja, namelijk […] / Nee<br><br>Nee / Ja, namelijk […]
Casus geschikt voor backward tracing?<br><br>*Indien ja: geef uitleg over de pilot*	Ja / Nee
Index wil meewerken aan backward tracing?	Nee, want […] / Ja, evt. afspraak gemaakt om contact op te nemen op [datum en tijd]
**Contacten in besmettelijke periode**<br>(LET OP: naam, geboortedatum, BSN, contactgegevens in apart event)	
Huisgenoten	[Aantal]
	[Per huisgenoot: relatie tot index, evt. laatste contact]
	Binnenshuis geïsoleerd leven is [wel/niet] mogelijk
Nauwe contacten	[Aantal]
	[Per contact/setting: relatie tot index, duur en afstand, datum laatste contact]
Overige contacten	[Aantal per groep/setting, datum]
Bezoek aan bijv. sportclub/restaurant/bioscoop in besmettelijke periode?	Nee / Ja, [namelijk setting, adres] op [datum]
Huisgenoot of nauw contact is prio contact	Nee / Ja, [namelijk...]
**Toestemming inlichten werkgever/huisarts/contacten/overige instanties**	Ja [welke personen?] / Nee, [want…]
Mag naam index worden genoemd	Ja / Nee
**Conclusie**	Index is wel/geen prio, want […]

**Beleid**	
Informatiemail(s) verstuurd naar index en verzocht deze door te sturen naar contacten.	Ja / Nee
Melding verstuurd via de CoronaMelder	Ja/ Nee, [want…]
Acties aangemaakt	Ja / Nee
Osiris-melding gemaakt	Ja / Nee
Index brengt huisarts telefonisch op de hoogte	Ja / Nee
Gegeven adviezen<br><br>*(LET OP: noteer ook welke specifieke adviezen je hebt gegeven voor contactpersonen (bijvoorbeeld als deze <18 jaar zijn en/of werkzaam zijn in de zorg)*	Indien symptomatisch:<br><br>Index blijft thuis tot 24 uur klachtenvrij en tot minimaal 7 dagen na de eerste ziektedag, dus tot ten minste [datum]<br><br>Indien asymptomatisch:<br><br>Index blijft tot 72 uur na testafname in isolatie. Huisgenoten en nauwe contacten gaan in diezelfde periode in quarantaine. 3 dagen na de test zal opnieuw telefonisch contact worden opgenomen met index
Besproken onderwerpen<br><br>*(LET OP: noteer alleen wat je benoemd heb inclusief evt. specifieke adviezen en beschrijf eventuele knelpunten die index voorziet)*	Niet naar buiten, school, winkel of werk gaan. Geen bezoek ontvangen. In eigen kamer blijven en apart slapen. Contact binnenshuis vermijden, 1,5 meter afstand houden. Niet knuffelen, zoenen en geen seks. Eigen servies, tandenborstel en aparte handdoeken gebruiken.
Actielijst BCO	
**Bijzonderheden**	Geen / Ja, [namelijk…]

FORMAT 2: BRONOPSPORING

**BWT: BRONOPSPORING**	
Wat is de EZD?	[datum]
Wat is de incubatieperiode?<br>*= EZD-8 t/m EZD-3*	[datum] – [datum]

Hoeveel nauwe contacten in de incubatieperiode?	
Hoeveel overige (risico)contacten de incubatieperiode?	
Hoeveel (risico)locaties in de incubatieperiode?	
**Contact 1**<br><br>• Naam en telefoonnummer.<br><u>Omschrijving contactmoment(en)</u><br><br>• Datum/data<br>• Aard van het contact<br>*Tijdsduur, afstand, risicocontact*<br>• Relatie tot index<br>*Bijv. vriend, huisgenoot, docent, etc.*<br>• Locatie<br>*Binnen/buiten, grootte, ventilatie, etc.*<br>• Risicocontact<br>*Contact had (vermoedelijk) klachten, hoog-risico contactmoment (hoesten/niezen/zoenen), contact had anderen in omgeving met klachten en/of positieve test*<br><u>Toestemming</u><br><br>• Index geeft toestemming om contact op te nemen?<br>• Mag naam van index genoemd worden?<br><br><u>Contact met VOB (na overleg met coördinator)</u><br><br>1. Heeft VOB klachten (gehad)?<br>2. Anderen in omgeving met klachten/positieve test?<br>3. Casus geschikt voor fase 2?	[Naam en telefoonnummer]<br><br>[Omschrijving]<br><br>Index geef [wel/geen] toestemming om contact op te nemen met VOB.<br>Naam van index mag [wel/niet] genoemd worden.<br><br>1. Nee / Ja, namelijk […] van [datum] tot [datum/heden]<br>2. Nee / Ja, namelijk […]<br>3. Nee, want […] / Ja, want […]
**Contact 2**<br><br>• Naam en telefoonnummer.<br><u>Omschrijving contactmoment(en)</u><br><br>• Datum/data<br>• Aard van het contact<br>*Tijdsduur, afstand, risicocontact*	[Naam en telefoonnummer]<br><br>[Omschrijving]

• Relatie tot index<br>*Bijv. vriend, huisgenoot, docent, etc.*<br>• Locatie<br>*Binnen/buiten, grootte, ventilatie, etc.*<br>• Risicocontact<br>*Contact had (vermoedelijk) klachten, hoog-risico contactmoment (hoesten/niezen/zoenen), contact had anderen in omgeving met klachten en/of positieve test*<br><u>Toestemming</u><br><br>• Index geeft toestemming om contact op te nemen?<br>• Mag naam van index genoemd worden?<br><br><br><u>Contact met VOB (na overleg met coördinator)</u><br><br>1. Heeft VOB klachten (gehad)?<br>2. Anderen in omgeving met klachten/positieve test?<br>3. Casus geschikt voor fase 2?	Index geef [wel/geen] toestemming om contact op te nemen met VOB.<br>Naam van index mag [wel/niet] genoemd worden.<br><br><br><br>1. Nee / Ja, namelijk […] van [datum] tot [datum/heden]<br>2. Nee / Ja, namelijk […]<br>3. Nee, want […] / Ja, want […]
**INDIEN MEERDERE CONTACTEN: KOPIEER DE BOVENSTAANDE REGELS EN PLAK HIER**	
**Risicolocatie 1**<br><br>• Naam locatie<br>• Datum/data van bezoek<br>• Soort locatie (bijv. supermarkt, werk, sportschool, restaurant)<br>• Omschrijving omstandigheden (ruimte, grootte, aantal mensen, ventilatie, etc.)	[Omschrijving]<br><br>Inschatting: [wel/niet] locatie van besmetting, want […]
**Risicolocatie 2**<br><br>• Naam locatie<br>• Datum/data van bezoek<br>• Soort locatie (bijv. supermarkt, werk, sportschool, restaurant)<br>• Omschrijving omstandigheden (ruimte, grootte, aantal mensen, ventilatie, etc.)	[Omschrijving]<br><br>Inschatting: [wel/niet] locatie van besmetting, want […]

**INDIEN MEERDERE RISICOLOCATIES: KOPIEER DE BOVENSTAANDE REGELS EN PLAK HIER**	
**Volgorde meest waarschijnlijke naar minst waarschijnlijke vermoedelijke bron(locatie)**<br>*Alleen in geval van meerdere bron(locaties)*	1. […]<br>2. […]<br>3. […]

Tijdsduur BWT: [aantal] minuten

FORMAT 3: VERMOEDELIJKE BRON (VOB)

**BWT: VERMOEDELIJKE BRON**	
**Deel 1**<br>*Gesprek index*	
Naam<br><br>*BSN-nummer en geboortedatum*	
Contactgegevens<br><br>Telefoonnummer<br><br>E-mailadres	
Reden vermoedelijke bron?	*Bijv. nauw contact of risicocontact*
Omschrijving contactmoment(en)<br><br>• Datum/data<br>• Aard van het contact<br>*Tijdsduur, afstand, risicocontact*<br>• Relatie tot index<br>*Bijv. vriend, huisgenoot, docent, etc.*<br>• Locatie<br>*Binnen/buiten, grootte, ventilatie, etc.*<br>• Risicocontact<br>*Contact had (vermoedelijk) klachten, hoog-risico contactmoment (hoesten/niezen/zoenen), contact had anderen in omgeving met klachten en/of positieve test*	[Omschrijving]<br><br>Inschatting: [wel/niet] VOB, want […]
Bijzonderheden	
**Deel 2**<br>*Gesprek VOB*	

Heeft VOB symptomen (gehad)?<br><br>*Zo ja, beschrijf zo nauwkeurig mogelijk het beloop*	Ja, namelijk [omschrijving klachten] vanaf [EZD] / Nee<br><br>Hersteld: ja, sinds [datum] / nee / n.v.t.<br>Restklachten: ja, namelijk [...] / nee / n.v.t.
Contact(en) in de omgeving met klachten en/of positieve test in afgelopen 2 weken?<br><br>*Zo ja, omschrijf per contact zo nauwkeurig mogelijk datum en (aard van) het contact, wanneer dit contact ziek is geworden en/of wat datum van positieve test was*	Nee / Ja, [omschrijving per contact]
Recent getest of op dit moment test ingepland?	Nee / Ja, op [datum/data] [positief/negatief] getest / Ja, test gepland op [datum]<br><br>Indien wel klachten en niet laten testen: [reden]
**Deel 3**<br>*Contactinventarisatie*	
Nauwe contacten	[aantal] nauwe contacten<br><br>Nauw contact 1: [naam] + [contactgegevens] + [omschrijving contactmoment]<br><br>Nauw contact 2: [naam] + [contactgegevens] + [omschrijving contactmoment]<br><br>…
Locaties	[aantal] locaties<br><br>Locatie 1: [naam] + [adres] + [omschrijving bezoek]<br><br>Locatie 2 [naam] + [adres] + [omschrijving bezoek]
**Deel 4**<br>*Adviezen aan VOB*	
Advies testen	Ja / Nee, want […]
Isolatieadvies	Ja tot ten minste [datum en/of 24 uur klachtenvrij] / Nee, want […]

Tijdsduur BWT: [aantal] minuten

FORMAT 4: BRONCONTACT

BWT: BRONCONTACT	
**Deel 1**	
Wie is de VOB?	[HP-nummer]
Laatste contact met VOB	[datum]
Omschrijving contactmoment(en)<br><br>• Datum/data<br>• Aard van het contact<br>*Tijdsduur, afstand, risicocontact*<br>• Locatie<br>*Binnen/buiten, grootte, ventilatie, etc.*	
Heeft BC symptomen (gehad)?<br><br>*Zo ja, beschrijf zo nauwkeurig mogelijk het beloop*	Ja, namelijk [omschrijving klachten] vanaf [EZD] / Nee<br><br>Hersteld: ja, sinds [datum] / nee
Recent getest of op dit moment test ingepland?	Nee / Ja, op [datum/data] [positief/negatief] getest / Ja, test gepland op [datum]<br><br>Indien wel klachten en niet laten testen: [reden]
Advies testen	Ja / Nee, want [...]
Isolatieadvies	Ja tot ten minste [datum en/of 24 uur klachtenvrij] / Nee, want [...]
**Deel 2**	
**CBC 1**	[Naam] + [telefoonnummer]
Laatste contactmoment	[datum]: [omschrijving]
Heeft BC symptomen (gehad)?<br><br>*Zo ja, beschrijf zo nauwkeurig mogelijk het beloop*	Ja, namelijk [omschrijving klachten] vanaf [EZD] / Nee<br><br>Hersteld: ja, sinds [datum] / nee
Gegeven (quarantaine)advies	[Advies]<br>CBC is bereid dit advies [wel/niet] te gaan opvolgen
**INDIEN MEERDERE CBC: KOPIEER DE BOVENSTAANDE REGELS EN PLAK HIER**	

Tijdsduur BWT: [aantal] minuten

BRONDOSSIER AANMAKEN

1. **New.**
2. **New case.**
    - BSN invullen, add as new person
    - Telefoonnummer en email toevoegen
3. **Assesment new case**
    - Covid-19
    - Probable
4. **Diagnostic notes**
    - PILOT BACKWARD TRACING
5. **Melding aan GGD**
    - Niets invullen
6. **Key details**
    - Date onset (datum eerste klachten): EZD (indien bekend) / Onbekend
    - Principal contextual settings: onbekend
7. **Administration**
    - Casemanager: Arts workload
    - Investigating officer: SVK workload
8. **GP Details**
    - Next
9. Plak het format 'VOB' in een Event.
10. Maak een actie aan volgens de juiste stap in deze werkinstructie.

DOORVERBINDEN AFSPRAKENLIJN

Het interne nummer van de afsprakenlijn: 38031

Het externe nummer van de afsprakenlijn: 010 4438100

IN DE WACHT ZETTEN

De opties voor in de wacht zetten en doorverbinden zie je alleen op het display tijdens een gesprek.

- Kies tijdens het gesprek de toets <IN WACHT>
- Toestel uit de wacht halen doe je door nogmaals op <IN WACHT> te drukken.

DOORVERBINDEN

1. Kies tijdens het gesprek de toets onder <Doorv.>

2. Bel het gewenste nummer (zie ook onder kopje 'Bellen') en vraag of je kunt doorverbinden. Afhankelijk van het antwoord:

    - Kies de toets onder <volt.> om daadwerkelijk door te verbinden.

    - Kies de toets onder <annul.> om het 2e gesprek te beëindigen en terug te gaan naar het eerste gesprek.

    - Kies de toets onder <beeind.> om het 2e gesprek te beëindigen en een ander nummer te kunnen bellen om naar door te verbinden.

N.B. Als degene naar wie je (intern) probeert door te verbinden in gesprek is kun je dat niet zien of horen, de telefoon gaat gewoon over. Dit komt doordat de telefoons meerdere lijnen tegelijk kunnen ontvangen. Bij degene naar wie je probeert door te verbinden is er wel een kort geluidssignaal te horen en gaat er een lampje knipperen als er een tweede lijn binnenkomt. Na het doorverbinden is er geen korte pauze van 30sec maar is je lijn direct beschikbaar voor inkomende oproepen.

Let op: Doordat er geen 30 sec. pauze is tussen doorverbinden en de volgende wachtende op lijn kan je in een korte periode verschillende stemmingen aan de telefoon krijgen.

CONTACTDOSSIER AANMAKEN

Let op: het aanmaken van het contactdossier gebeurt op dezelfde wijze als bij regulier BCO. In het kader van deze pilot hoeft echter niet alles op dezelfde manier te worden ingevuld. Hieronder staat dan ook een aangepaste versie die alleen voor de pilot gebruikt dient te worden.

Controleren of contactdossier al bestaat

Check eerst of het contact al bestaat in HP Zone. Via 'Look-up' bovenin je scherm kun je zoeken op naam, BSN of 06-nummer.

1. Ga naar Contacts in het rechterpaneel klik op: assign an existing Contact.
2. Je krijgt nu een lijst met alle open contacten te zien, klik de juiste persoon aan.
3. Geef daarna aan wat de relatie van het contact met de case was en klik op OK.

NB: Tijdens 'Lean BCO' worden er in principe geen contactdossiers aangemaakt (behalve bij scholing). Wanneer er toch een contactdossier bestaat, controleer de gegevens en plak het format 'Broncontact' in een Event.

Aanmaken nieuw contactdossier

1. Ga naar *Contacts* in het rechter paneel en klik de juiste optie aan:

    a. Huisgenoten: *Add a new household contact.*

    b. Nauwe contacten: *Add a new contact*

2. Je komt nu automatisch in het personal details veld. Vul de personal details in aan de hand van het BSN-nummer. Klik op *Lookup on BSN*, als dit de goede persoon is klik je op *Update field*. Indien het BSN-nummer (nog) niet bekend is maak je een dossier aan op basis van naam en evt. geboortedatum (de rode velden zijn de verplichte velden). Je kunt de gegevens dan later aanvullen. Klik vervolgens op search.
3. Je komt vervolgens in een scherm waarin de eventuele matches vermeld staan, klik hier de juiste aan.
    - Indien er wel matches zijn: controleer of dit echt om dezelfde persoon gaat en controleer vervolgens waarom iemand al een dossier heeft. Neem afhankelijk hiervan de juiste stappen.
    - Indien er geen matches zijn kom je in het onderstaande scherm, klik in dit geval op continue, er wordt dan een nieuw dossier aangemaakt.
4. Je komt nu opnieuw in het *Personal Details*-scherm, ditmaal is deze uitgebreider. Vul deze in met alle bekende informatie, de volgende informatie moet in ieder geval geregistreerd worden. Klik hierna op save.
    a. Reference name
    Indien deze nog leeg is → vul initialen en achternaam in

Voeg achter de naam tussen haakjes in of persoon huisgenoot of nauw contact is. NB: hier niet invullen of dit partner/broer/kind etc. is, dat volgt later.
   b. Geboortedatum
   c. Geslacht
   d. Telefoonnummer
   e. E-mailadres
   f. Alle andere bekende informatie
5. Je komt vervolgens in een scherm waar je moet aangeven wat de relatie is van de persoon tot de index. Vink de juiste relatie aan en klik op OK:
6. Je wordt nu gevraagd naar wat voor contact het is, klik hier de juiste aan.
7. In het volgende scherm klik je de volgende onderdelen aan.
   a. *Key details*
      i. Additional comments on the nature of the contact: PILOT BWT
      - Degree of contact
      - Current location
   b. Administration:
      - *Case manager*: vul hier in "Arts workload"
      - *Investigating officer*: vul hier in SVK workload.
8. Je komt vervolgens automatisch in de *GP-practice* (=huisartsenpraktijk), deze hoeft niet ingevuld te worden, klik op *next*.
9. Je hebt nu een dossier aangemaakt. Het format 'Broncontact' kan in het Event worden geplakt.
10. Maak de juiste acties aan.

EVALUATIEFORMULIER BCO-MEDEWERKER

Het is erg belangrijk om de ervaring van de BCO-medewerker te evalueren alvorens er uitspraak kan worden gedaan over het succes van de pilot.

Vul onderstaand formulier zo nauwkeurig mogelijk in. Het gaat om de **gemiddelde** indruk die je hebt gekregen na het doen van BWT. Uitzonderingen hierop kunnen worden vermeld in het vak 'Toelichting'. Geef ieder criterium een cijfer van 1 t/m 5. Voor ieder criterium geldt dat 1 'helemaal niet/slecht/oneens' en 5 'helemaal wel/goed/eens' betekent. Graag horen we ook of je nog ideeën hebt voor de procesverbetering.

Naam	Aantal weken BCO-ervaring	Datum
**Criterium**	**Evaluatie**<br>**1 2 3 4 5**	**Toelichting**
Algemeen concept BWT<br>• Moeite waard?<br>*Extra tijd t.o.v. winst*<br>• Geschikt als onderdeel regulier BCO?	o o o o o<br>o o o o o<br>o o o o o	
Medewerking contacten<br>• Prettig gesprek<br>• Bereid om tijd te investeren<br>• Bereid om adviezen op te volgen	o o o o o<br>o o o o o<br>o o o o o	
Verkregen informatie van contacten<br>• Snelheid/efficiëntie<br>*Bijv. was het nodig om terug te bellen?*<br>• Nauwkeurigheid<br>• Betrouwbaarheid	o o o o o<br>o o o o o<br>o o o o o	
Werkinstructie<br>• Algemeen<br>• Formats		

• Excelsheet	o o o o o<br>o o o o o<br>o o o o o	
Geschiktheid registratie in HP Zone	o o o o o	
**Ideeën voor procesverbetering?** (bijv. werkinstructie, formats, hulpmiddelen tijdlijn)		

Supplementary table **S3**, Outcomes of backward contact tracing of SARS-CoV-2 positive index cases by public health service Rotterdam-Rijnmond, February and March 2021

**Outcome**	**n**	**%**
Routine contact tracing interviewing index		
Total index cases	7,448	100%
Potential source registered case in PHS database	3,497	47%
Potential source completely unknown	2,200	30%
Potential source known to the index, but not in PHS database	979	13%
Missing data	772	10%
Dedicated backward tracing team interviewing index		
Total index cases interviewed	979	100%
Potential source found in PHS database by index providing further personal details	431	44%
Only a potential source location known	333	34%
At least 1 potential source identifiable, not known to PHS database	62	6%
Index no consent	58	6%
Other	95	10%
Dedicated backward tracing team interviewing potential sources		
Total potential sources interviewed	133	100%
Asymptomatic	71	53%
Mismatch infectious period with index	20	15%
Not reachable by phone	13	10%
Waiting for test or to be contacted because of outbreak investigation	13	10%
No cooperation	7	5%
Potential source found in PHS database by source providing further personal details	5	4%
Tested for SARS-CoV-2	4	3%

Reasons reported by the contact tracing team to exclude from backward contact tracing: index reported to not have (close) contacts during the period prior to disease onset; the index reported to not have contacts with symptoms indicative of COVID-19; the index reported to have only anonymous contacts for which the index case could not provide contact details.

Supplementary material **S4**, questionnaires backward contact tracing

During the study each index case was interviewed using the standard (forward) contact tracing questionnaire. Topics discussed during this interview are shown below. For the purpose of backward contact tracing, additional questions were asked to the index case, potential sources (G0-contacts) and their contacts (G1- and G2-contacts).

Questionnaire index case (forward contact tracing)

Clinical data

- Date of test administration
- Reason for testing
- First day symptoms (FDS)
- Type of symptoms
- Diseases/conditions (e.g., overweight)
- Weakened immune system due to illness or medication
- Pregnancy or recent birth
- Name of family doctor

Prioritization

- Current place of residence
- Recent hospitalization
- Involvement of emergency services (e.g., police, ambulance)
- Receiving home care
- Healthcare institution visited (e.g., hospital, care home)
- Visit or visit GP (post)
- Stay abroad or flown/use of group transport
- Visitor/employee school or day-care

Profession

- Organization, location, department
- Function
- Last working day
- Worked during contagious period
- Close contacts at work
- Number of other contacts
- Colleagues with symptoms
- Measures in the workplace
- Unrest at work
- Organization contact person

- Employer already informed
- Incubation period
- Close contacts or risk contacts during this period
- Risk location
- Possible part of cluster/situation/outbreak

- Housemates
- Close contacts
- Other contacts
- Visit to, for example, a sports club/restaurant/cinema during an infectious period?
- Consent to inform employer/ GP/contacts/other authorities

Contacts in contagious period

Questionnaire index case (backward contact tracing)

Source tracing

- Known source in electronic database PHS
- Potential source tracing (if source unknown)
  - Incubation period
  - Close contacts or risk contacts during this period
  - Nature of contact
  - Risk location
  - Possible part of cluster/situation/outbreak
  - Case suitable for backward tracing
    - If yes: explain the pilot
  - Index wants to participate in backward tracing

Additional questionnaire potential source case (G0-contact)

Clinical data

- Does G0-contact have (or have had) symptoms
- Type of symptoms if applicable
- Contact(s) in the area with complaints and/or positive test in the past 2 weeks
- Recently tested or currently testing scheduled

Contact tracing

- Close contacts during contagious period
- Locations during contagious period

Advices

- Test advice
- Isolation advice

Additional questionnaire G1-contact

Source tracing

- Name of potential source
- Last contact with potential source
- Description of contact moment(s)
    - Date(s)
    - Nature of the contact
    - Location

Clinical data

- Does G1-contact have (or have had) symptoms
- Type of symptoms if applicable
- Recently tested or currently testing scheduled

Advices

- Test advice (if symptoms)
- Insulation advice (if symptoms)

Additional questionnaire G2-contact

Clinical data

- Does G1-contact have (or have had) symptoms
- Type of symptoms if applicable
- Recently tested or currently testing scheduled

Advices

- Quarantine advice

Supplementary figure **S5**, example backward contact tracing

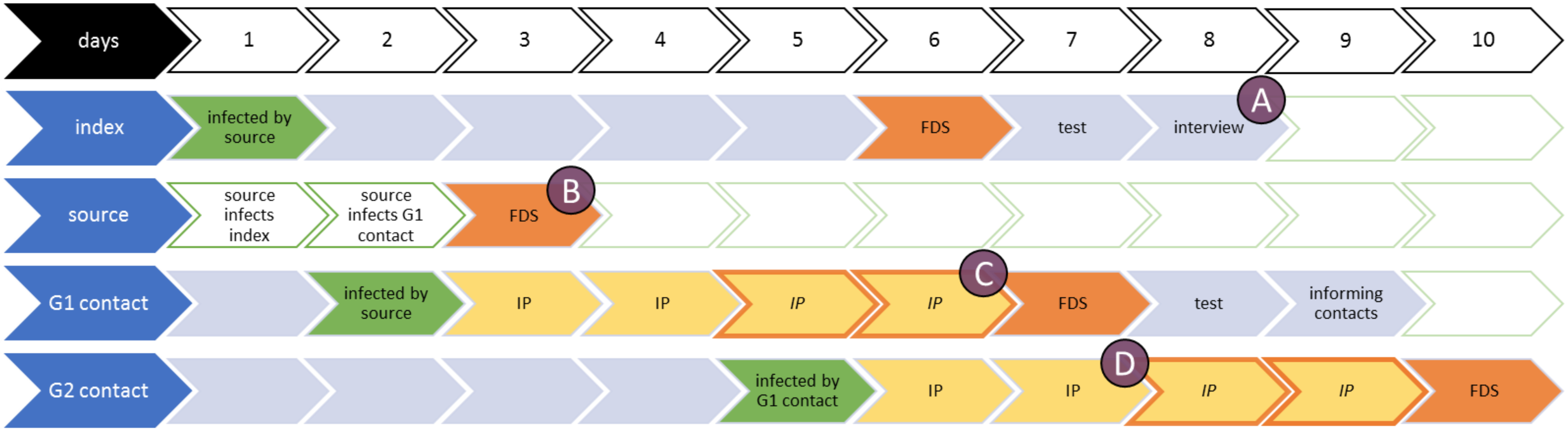

Example of viral transmission between source, generation-1 and generation-2 contacts.

FDS: first day of symptoms; G0: generation 0; G1: generation 1; G2: generation 2; IP: incubation period.

The arrows outlined in orange represent the period that an infected person is already infectious, before onset of disease.

By identifying the potential source of the index case and quarantining the generation 1 and generation 2 contacts of this source, the viral transmission chain may be interrupted. A = the index case is being interviewed by a contact tracer. B = information from the index case leads to a potential source with symptom onset a few days earlier. C = by interviewing the source case, generation-1 contacts can be identified who are potentially also infected by the source. D = by interviewing generation-2 contacts who are potentially infected by the generation-1 contact but have not yet become infectious, and advise them to quarantine, further spreading of SASR-CoV-2 could be prevented.
